# Identification of candidate genes and residues for improving nitrogen use efficiency in the N-sensitive medicinal plant *Panax notoginseng*

**DOI:** 10.1186/s12870-024-04768-4

**Published:** 2024-02-12

**Authors:** Zhu Cun, Xia Li, Jin-Yan Zhang, Jie Hong, Li-Lin Gao, Jing Yang, Su-Yun Ma, Jun-Wen Chen

**Affiliations:** 1 College of Agronomy & Biotechnology, Yunnan Agricultural University, Fengyuan Road, Panlong District, Kunming, 650201 China; 2 Key Laboratory of Medicinal Plant Biology of Yunnan Province, Yunnan Agricultural University, Kunming, 650201 China; 3 National & Local Joint Engineering Research Center on Germplasm Innovation & Utilization of Chinese Medicinal Materials in Southwestern China, Yunnan Agricultural University, Kunming, 650201 China

**Keywords:** Nitrogen deficiency, Nitrogen metabolism, Nitrogen use efficiency, Molecular docking, *Panax notoginseng*

## Abstract

**Background:**

Nitrogen (N) metabolism-related key genes and conserved amino acid sites in key enzymes play a crucial role in improving N use efficiency (NUE) under N stress. However, it is not clearly known about the molecular mechanism of N deficiency-induced improvement of NUE in the N-sensitive rhizomatous medicinal plant *Panax notoginseng* (Burk.) F. H. Chen. To explore the potential regulatory mechanism, the transcriptome and proteome were analyzed and the three-dimensional (3D) information and molecular docking models of key genes were compared in the roots of *P. notoginseng* grown under N regimes.

**Results:**

Total N uptake and the proportion of N distribution to roots were significantly reduced, but the NUE, N use efficiency in biomass production (NUEb), the recovery of N fertilizer (RNF) and the proportion of N distribution to shoot were increased in the N_0_-treated (without N addition) plants. The expression of N uptake- and transport-related genes *NPF1.2*, *NRT2.4*, *NPF8.1*, *NPF4.6*, *AVP*, proteins AMT and NRT2 were obviously up-regulated in the N_0_-grown plants. Meanwhile, the expression of *CIPK23*, *PLC2*, *NLP6*, *TCP20*, and *BT1* related to the nitrate signal-sensing and transduction were up-regulated under the N_0_ condition. Glutamine synthetase (GS) activity was decreased in the N-deficient plants, while the activity of glutamate dehydrogenase (GDH) increased. The expression of genes *GS1-1* and *GDH1*, and proteins GDH1 and GDH2 were up-regulated in the N_0_-grown plants, there was a significantly positive correlation between the expression of protein GDH1 and of gene *GDH1*. Glu192, Glu199 and Glu400 in PnGS1 and PnGDH1were the key amino acid residues that affect the NUE and lead to the differences in GDH enzyme activity. The 3D structure, docking model, and residues of *Solanum tuberosum* and *P. notoginseng* was similar.

**Conclusions:**

N deficiency might promote the expression of key genes for N uptake (genes *NPF8.1*, *NPF4.6*, *AMT*, *AVP* and *NRT2*), transport (*NPF1.2* and *NRT2.4*), assimilation (proteins GS1 and GDH1), signaling and transduction (genes *CIPK23*, *PLC2*, *NLP6*, *TCP20*, and *BT1*) to enhance NUE in the rhizomatous species. N deficiency might induce Glu192, Glu199 and Glu400 to improve the biological activity of GS1 and GDH, this has been hypothesized to be the main reason for the enhanced ability of N assimilation in N-deficient rhizomatous species. The key genes and residues involved in improving NUE provide excellent candidates for the breeding of medicinal plants.

**Supplementary Information:**

The online version contains supplementary material available at 10.1186/s12870-024-04768-4.

## Background

Nitrogen (N) is a limiting factor for crop yield. To meet the increasing demand for food from the growing global population, over 110 Tg of N fertilizer is applied annually to improve crop yields [1]. However, less than 40% of applied N fertilizer is absorbed by crops, the remaining N fertilizer is lost to the environment through processes such as volatilization, leaching, surface runoff, or microbial consumption [2, 3]. Hence, N use efficiency (NUE) in plants has to be improved [4]. NUE is a complex trait influenced by both genetic and environmental factors [5]. NUE mainly depends on how plants uptake inorganic N from the soil, assimilate nitrate (NO_3_^-^) and ammonium (NH_4_^+^) [6]. A better understanding of N uptake, transportation and assimilation within the plant is crucial for breeding for crops with high NUE and for the development of sustainable agriculture.

The low-affinity transport system (LATS) and the high-affinity transport system (HATS) ensure an efficient uptake over a wide range of external NO_3_^-^ concentrations [7]. Phosphorylation of a threonine residue, Thr101, lead to a switch of NRT1.1 from low- to high-affinity state [8]. The expression of NRT2/NPF initiates HATS in the N-deficient condition [9]. Overexpression of *OsNRT2.1* and *OsNRT2.3* in *Oryza sativa* has been found to improve NUE and yields under the N deficiency condition [10, 11]. NO_3_^-^ are degraded to NH_4_^+^, then NH_4_^+^ form amino acid through catalysis by N metabolism-related enzyme, as reflected by nitrite reductase (NiR), glutamine synthetase (GS) and glutamate dehydrogenase (GDH) [9]. Overexpression of *DvGS1/2* and *OsGS1* is positively associated with the biomass and NUE in the N-deficient *Dunaliella viridis*, *Arabidopsis thaliana* and *O. sativa* [12–14]. Meanwhile, the enhanced NO_3_^-^ signal-sensing and transduction pathways might promote N uptake and utilization, and thus improve NUE [15]. The increased expression of *NRT1.1*, *NRT2.1*, *NIA*, *NIR1*, and *GS2* in *NLP7*-overexpressing *A. thaliana* cloud improve the biomass and NUE under N stress [16]. Extensive reports are available on N-mediated regulation of enzymes and genes involved in N metabolism [17–19]. Significantly, Pro492 and Ser487 residue of NRT1.1 is important for NO_3_^-^ transport activity and NUE in *A. thaliana* [20]. Gln433 and Tyr512 residue play an important role in a comprehensive understanding of NPF genes for low N tolerance in *Setaria italica* [21]. Thus, dissecting the three-dimensional (3D) information and molecular docking models of key genes for N uptake, transportation and assimilation is important for improving plant NUE.

*Panax notoginseng* is a perennial rhizomatous medicinal plant and is also an N-sensitive species in the Araliaceae family. The application of N fertilizer in *P. notoginseng* cultivation can be as high as 337.5-450 kg·ha^-1^·year^-1^, which is two times the amount of crops like *Zea may*s and *O. sativa*, and four to five times the amount of *Nicotiana tabacum*. Excessive N fertilizer not only reduces NUE and increases production costs, but also causes continuous cropping barrier, severe field diseases, and quality decline in *P. notoginseng* [22]. NUE is significantly increased in two- and three-year-old *P. notoginseng* grown under the N deficient condition, and high N application significantly inhibits N uptake and NUE, and reduces biomass accumulation [23, 24]. It is worth noting that GS, GDH, and NR activity in the N-deficient *P. notoginseng* are higher than those in N-surplus groups (450 kg·ha^-1^), and the expression levels of *GS1*, *GDH1*, *NIR*, *NIA*, and *NPF* are up-regulated under the N deficiency levels [22]. However, it is still unknown about the molecular mechanisms underlying the low N tolerance in *P. notoginseng*. Although N uptake and NUE has been preliminarily studied in *P. notoginseng* under N regimes [22, 24], the underlying reason for the N regulation of NUE remain largely undetermined.

In this study, N uptake and utilization indicators and N metabolism-related enzymes and genes were analyzed in two-year-old *P. notoginseng* grown under N_0_ (without N addition), N_7.5_ (112.5 kg·ha^-1^, mild N deficiency), and N_15_ (225 kg·ha^-1^, normal N) condition. Key genes for N assimilation in response to N deficiency, coupled with the multi-omics analysis, 3D information and molecular docking models, were further identified. A systematic investigation was conducted on the expression of the genes and residues related to N uptake, transport, assimilation, NO_3_^-^ signal-sensing and transduction in the N-deficient plants. The present study would provide a valuable information for selecting candidate genes to improve NUE and further for investigating the function of N assimilation-related enzymes in the medicinal plants, such as *P. notoginseng*.

## Results

### The effect of N levels on Carbon(C) and N contents in *P. notoginseng*

N content of leaf and stem was significantly increased in the N_15_-treated plants (Table 1). There was no significant difference in N content between the N_7.5_ and N_15_ treatments, except for the stem (Table 1). C content in all tissues showed no significant difference among treatments (Table 1).

**Tabel 1:** Effects of nitrogen levels on N and organic carbon (C) content in *Panax notoginseng*

Variables	Different tissues	Nitrogen levels		
		N_0_	N_7.5_	N_15_
Nitrogen content (%)	Taproot	0.61 ± 0.02 b	1.37 ± 0.01 a	1.37 ± 0.01 a
	Rhizome	0.95 ± 0.07 b	2.01 ± 0.02 a	1.88 ± 0.04 a
	Fibrous root	1.30 ± 0.03 b	2.07 ± 0.05 a	2.04 ± 0.02 a
	Stem	0.59 ± 0.01 c	1.01 ± 0.01 b	1.09 ± 0.03 a
	Leaf	1.52 ± 0.03 b	2.42 ± 0.05 a	2.52 ± 0.01 a
Organic carbon content (%)	Taproot	43.66 ± 0.17 a	42.27 ± 0.90 a	43.99 ± 0.26 a
	Rhizome	42.93 ± 0.43 a	41.03 ± 0.55 a	44.65 ± 2.48 a
	Fibrous root	40.55 ± 0.35 a	38.66 ± 0.60 a	39.06 ± 1.21 a
	Stem	35.18 ± 0.35 a	35.96 ± 0.51 a	36.48 ± 1.48 a
	Leaf	41.24 ± 0.37 a	42.38 ± 0.49 a	42.80 ± 0.57 a

Different letters in the same row indicate significant difference (*P* < 0.05), values are means ± SD (*n* = 3)

### Responses of N uptake and utilization to N regimes

**Fig. 1:**
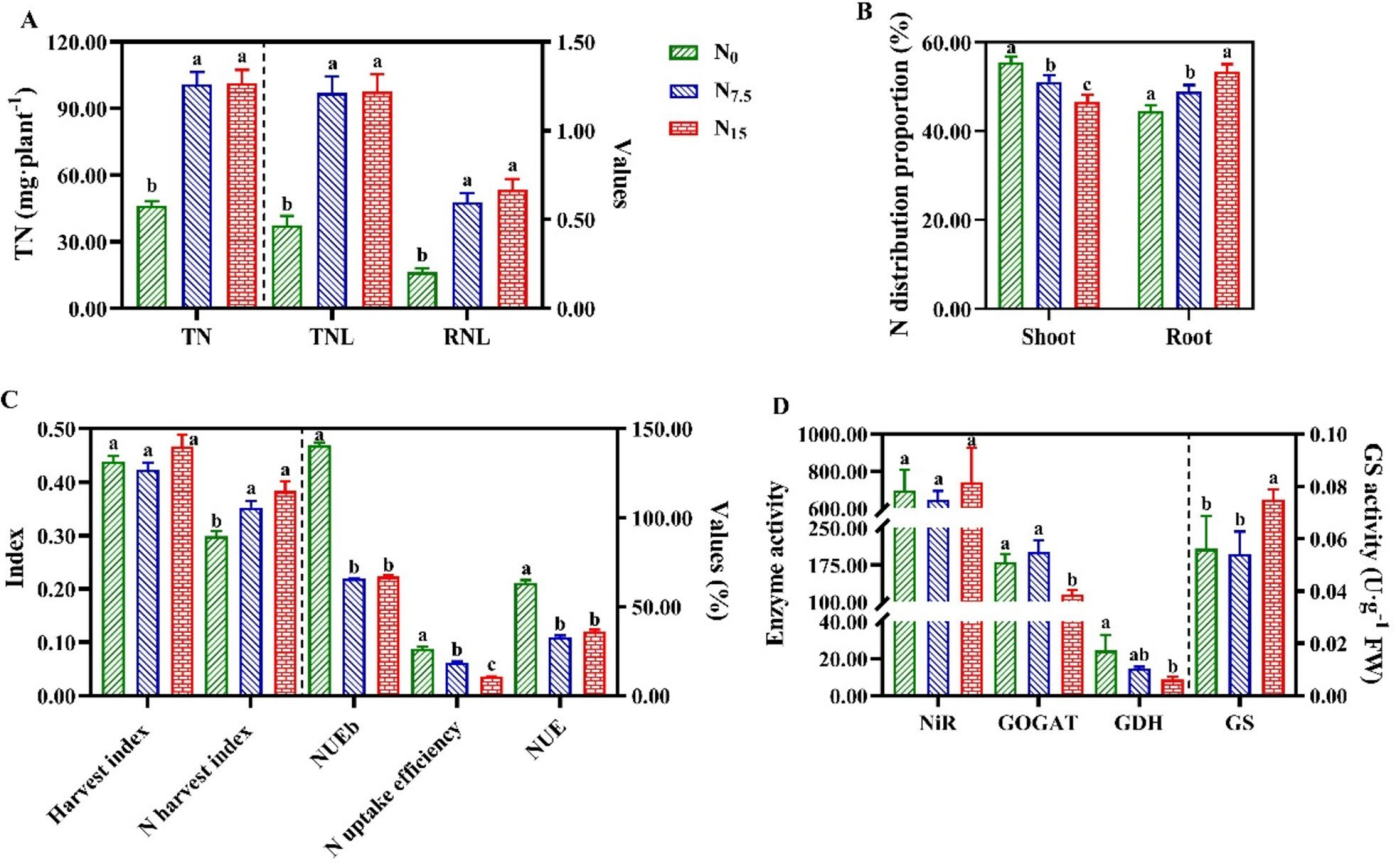
Effects of nitrogen levels on N uptake, use and N metabolism-related enzymes activity in *Panax notoginseng.* **A** TN is the total N uptake (mg·plant^-1^), TNL is the total N uptake per unit root length (mg·cm^-1^), RNL is the root N content per unit root length (mg·cm^-1^); **B** Proportion of N distribution to shoot and root (%); **C** Harvest index, N harvest index, NUEb is N use efficiency in biomass production (g·DW g^-1^·N), N uptake efficiency (kg·kg^-1^), NUE is the N use efficiency (kg·kg^-1^); **D** Nitrite reductase (NiR, μmol·h^-1^·g^-1^ FW), glutamine synthetase (GS, U·g^-1^ FW), glutamate synthetase (GOGAT, nmol Glu·min^-1^·g^-1^ FW), glutamate dehydrogenase (GDH, nmol·min^-1^·g^-1^ FW). Green represents N_0_, blue represents N_7.5_, red represents N_15_. Values for each point were means ± SD (*n* = 3). Significant differences are indicated by letters (ANOVA; *P* < 0.05)

Total N uptake (TN), total N uptake per unit root length (TNL), and root N content per unit root length (RNL) were significantly decreased in the N_0_-grown plants compared with the N_7.5_- and N_15_-grown plants (Fig. 1A). A proportion of N distribution to shoot was decreased with increasing N supply, while the proportion of N distribution to root was increased (Fig. 1B). There was no significant difference in the harvest index among N regimes (Fig. 1C). The maximum values of N use efficiency in biomass production (NUEb), N uptake efficiency and NUE were recorded in the N_0_-grown plants (Fig. 1C). N partial factor productivity (NPFP) and N agronomic efficiency (NAE) were no significantly different between the N_7.5_ and N_15_ treatments (Table 2). N contribution rate (NCR) was significantly increased in the N_15_-treated plant, which the recovery of N fertilizer (RNF) was decreased (Table 2).

**Table 2:** Effects of N levels on N agronomic efficiency in *P. notoginseng*

Variables	Nitrogen levels		
	N_0_	N_7.5_	N_15_
NPFP (kg·kg^-1^)	—	8.86 ± 3.03 a	4.88 ± 1.93 a
NAE (kg·kg^-1^)	—	1.54 ± 3.46 a	1.22 ± 2.15 a
NCR (%)	—	4.31 ± 53.07 b	10.34 ± 49.91 a
RNF (%)	—	33.66 ± 8.72 a	16.90 ± 4.86 b

*NPFP* N partial productivity, *NAE* N agronomic efficiency, *NCR* N contribution rate, *RNF* Recovery of N fertilizer

Values followed by different letters are significantly different at *P* < 0.05

### DIA-proteome quality control and differential protein analysis

A total of 53078 peptides and 8042 proteins were identified in the proteome (Figure S2A). The statistical analysis of the number of peptides per protein showed that most proteins had 1-5 or >10 peptide segments (Figure S2B). 7085, 3053, and 5902 proteins were annotated in the GO, KEGG KOG database among the 8042 proteins, respectively (Figure S3A). There were 65 up-regulated and 247 down-regulated proteins in the N_0_ vs N_7.5_ group, and 84 up-regulated and 269 down-regulated proteins in the N_0_ vs N_15_ group (|log_2_(1.5)| ≈ 0.58, Figure S3B). For GO enrichment analysis, The differentially expressed proteins were mainly annotated to processes such as "metabolic process", "cellular process", "cell part" and "response to stimulus" (Figure S4). KEGG pathway analysis revealed that the differentially expressed proteins in the N_0_ vs N_7.5_ and N_0_ vs N_15_ comparison groups were mainly enriched in metabolic pathways such as "biosynthesis of secondary metabolites "and "carotenoid biosynthesis", "N metabolism" (Figure S5). As shown in Figure S6, trend 1, trend 0, and trend 6 were the three largest clusters, with 270, 81, and 65 differentially expressed proteins, respectively. Trend 0 were significantly up-regulated under the N_0_ condition compared with the N_7.5_ and N_15_ conditions (Figure S6). The functions of Trend 0 mainly included pathways such as "metabolic pathways", "starch and sucrose metabolism", and "N metabolism" (Figure S7).

### Responses of N uptake, transport, and assimilation to N regimes

**Fig. 2:**
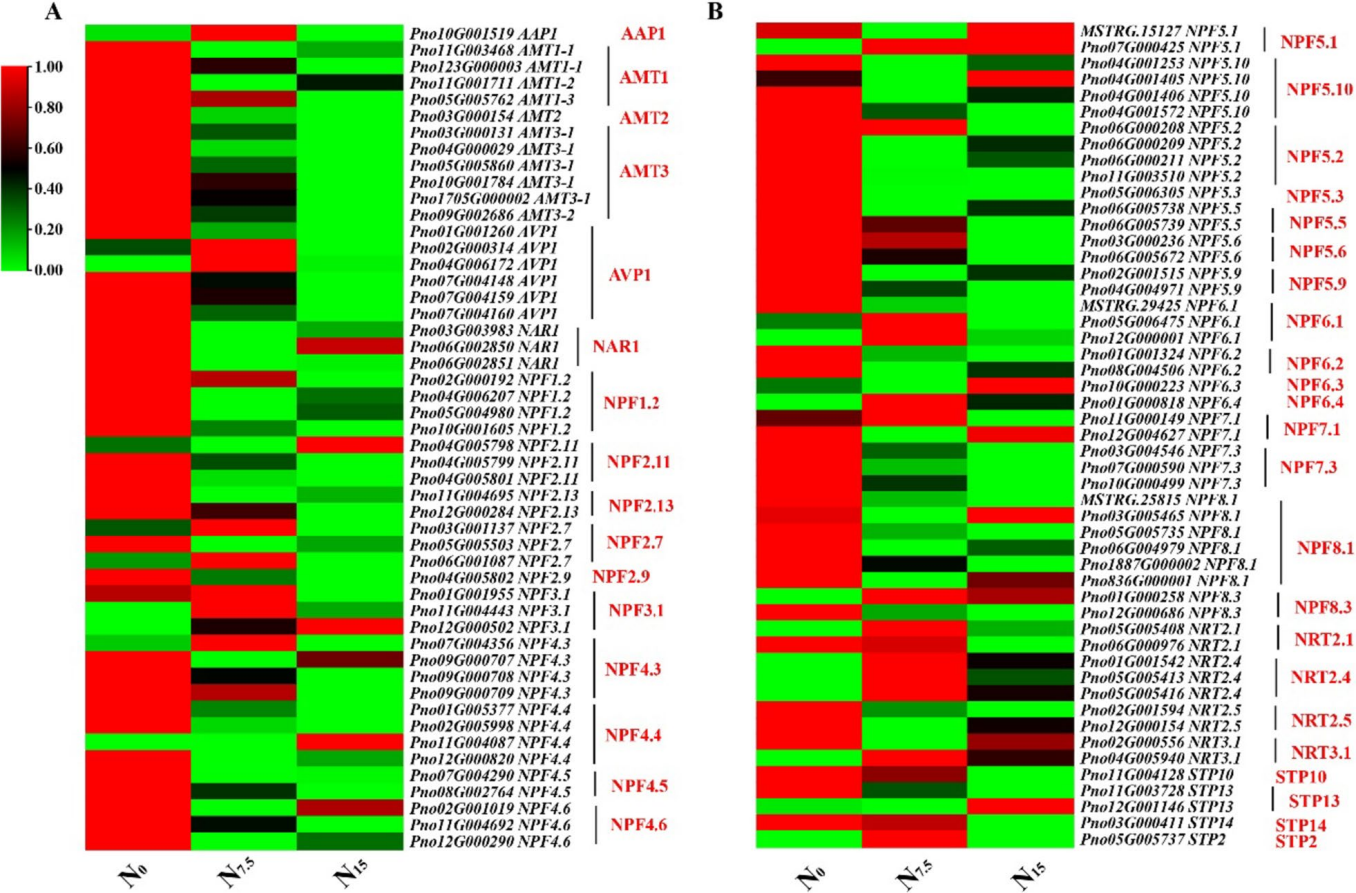
Response of N uptake- and transport-related genes to N levels. Average genes intensity is color key scale according the scale in the middle upper part

**Fig. 3:**
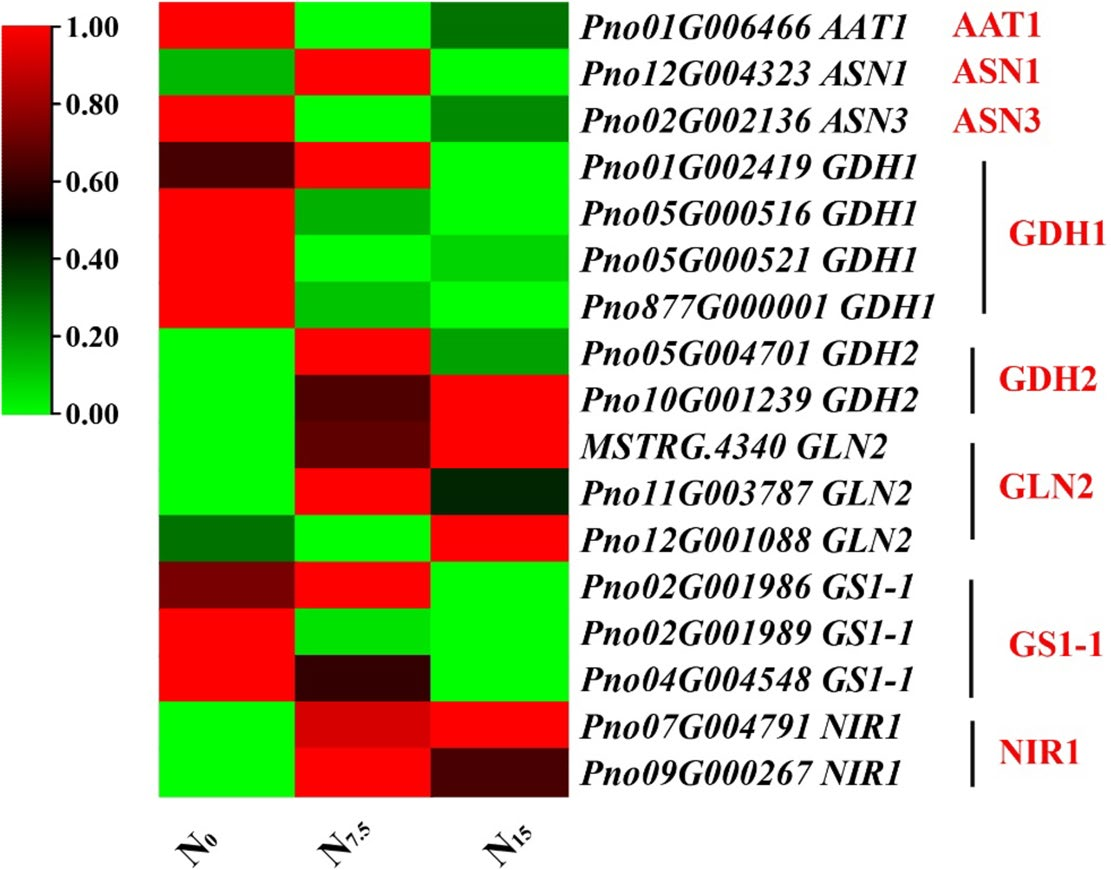
Response of N assimilation-related genes to N levels. Average genes intensity is color key scale according the scale in the middle upper part

**Fig. 4:**
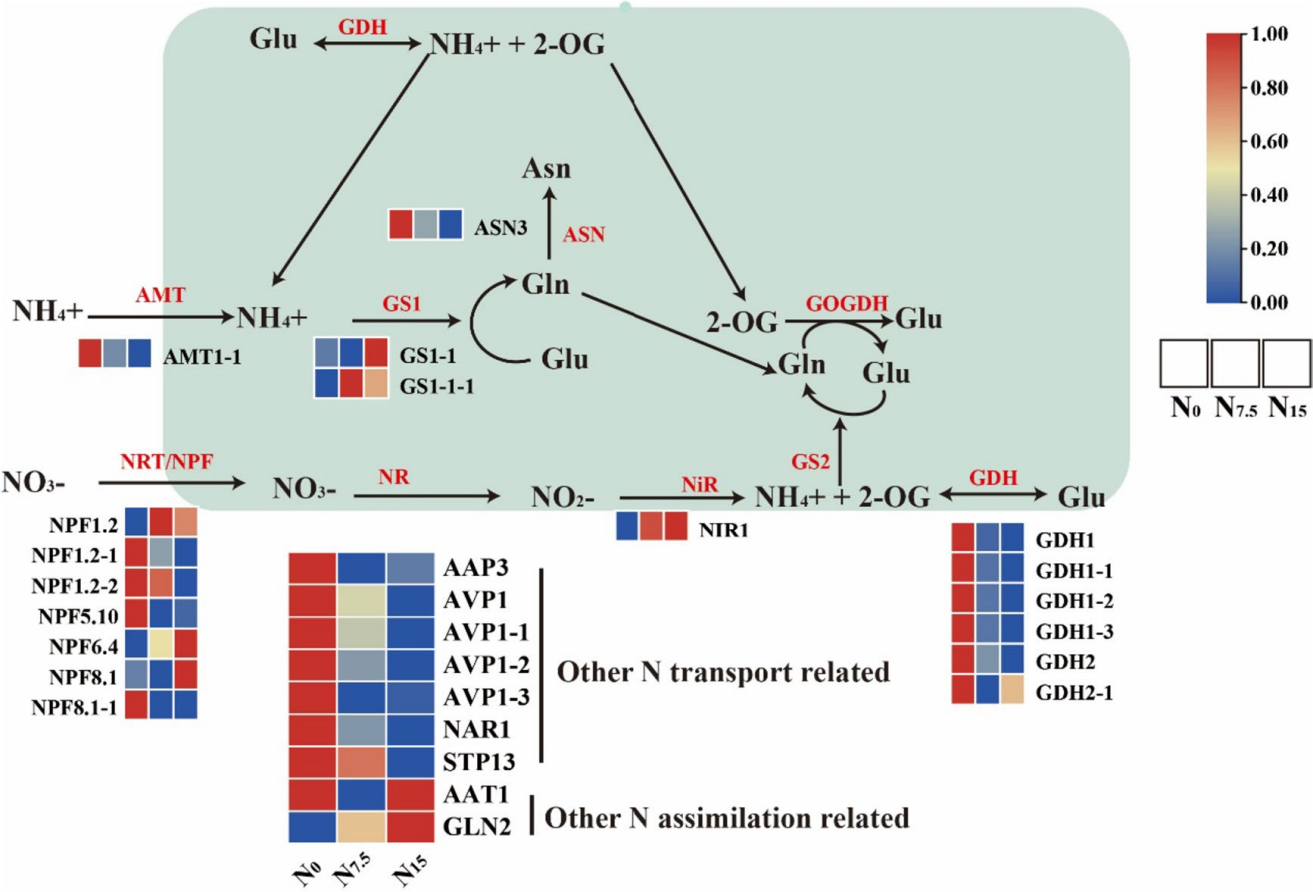
Response of N uptake-, transport- and assimilation-related proteins to N levels. The pathway map was prepared by using KEGG PATHWAY Database. With each box, each column is different nitrogen treatment (from left to right: N_0_, N_7.5_ and N_15_) as shown in the middle upper part. Average proteins intensity is color key scale according the scale in the middle upper part

The expression of NH_4_^+^ transport-related genes *AMT1-1, AMT1-2, AMT1-3, AMT2, AMT3-1, AMT3-2*, *AVR1* and protein AMT1-1 were up-regulated in the N_0_-grown plants compared with the N_7.5_- and N_15_-grown plants (Figs. 2 and 4). The expression of NO_3_^-^ uptake and transport-related genes *NPF1.2, NPF2.11, NPF2.13, NPF2.9, NFP4.5, NPF4.6, NPF5.2, NPF5.3, NPF5.5, NPF5.6, NPF5.9, NPF6.2, NPF7.3, NPF8.1, NRT2.5, STP10*, *STP14* and proteins NPF1.2, NPF5.10, NPF8.1, AAP3, AVP1, NAR1 and STP13 were significantly up-regulated under the N_0_ condition compared with the N_7.5_ and N_15_ conditions (Figs. 2 and 4). The expression of N uptake and transport-related genes were down-regulated in the N_15_-grown plants (Fig. 2). N assimilation-related genes (such as *AAT1, ASN3, GDH1*, and *GS1-1*) and proteins (such as GDH1, GDH2, ASN3 and AAT1) were up-regulated in the N_0_-grown plant compared with the N_7.5_- and N_15_-grown plants (Fig. 3), which protein GS and NiR were down-regulated (Fig. 4). The gene expression trends of *AAT1*, *ASN3*, *GDH1*, *AMT2*, *AVP1*, *NPF1.2*, *NPF4.6*, *NPF8.1* and *NPF 2.4* were verified by Real-time quantitative polymerase chain reaction (qRT-PCR) were consistent with the results of root RNA-seq data (Figures S8-S9).

### Responses of N metabolism-related enzymes activity to N regimes

There was no significant difference in the activity of nitrite reductase (NiR) among N regimes (Fig. 1D). The maximum and minimum values of glutamine synthetase (GS) and glutamate synthetase (GOGAT) activity were recorded in the N_15_-grown plants, respectively (Fig. 1D). The activity of glutamate dehydrogenase (GDH) significantly decreased with increasing N supply (Fig. 1D).

### Responses of NO_3_^-^ signal-sensing and transduction to N regimes

**Fig. 5:**
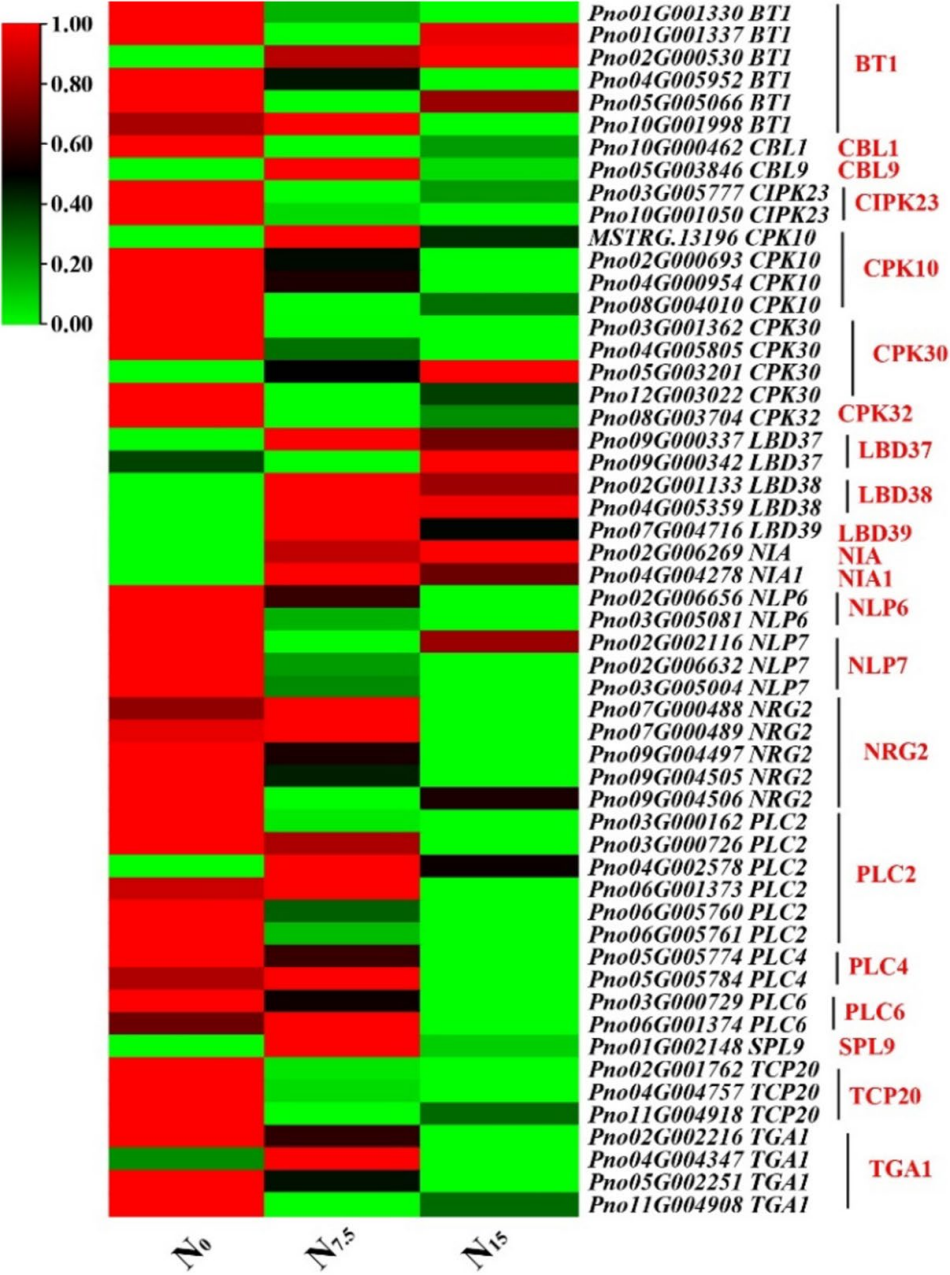
Response of nitrate signal-sensing and transduction pathway-related genes to N levels. Average genes intensity is color key scale according the scale in the middle upper part

**Fig. 6:**
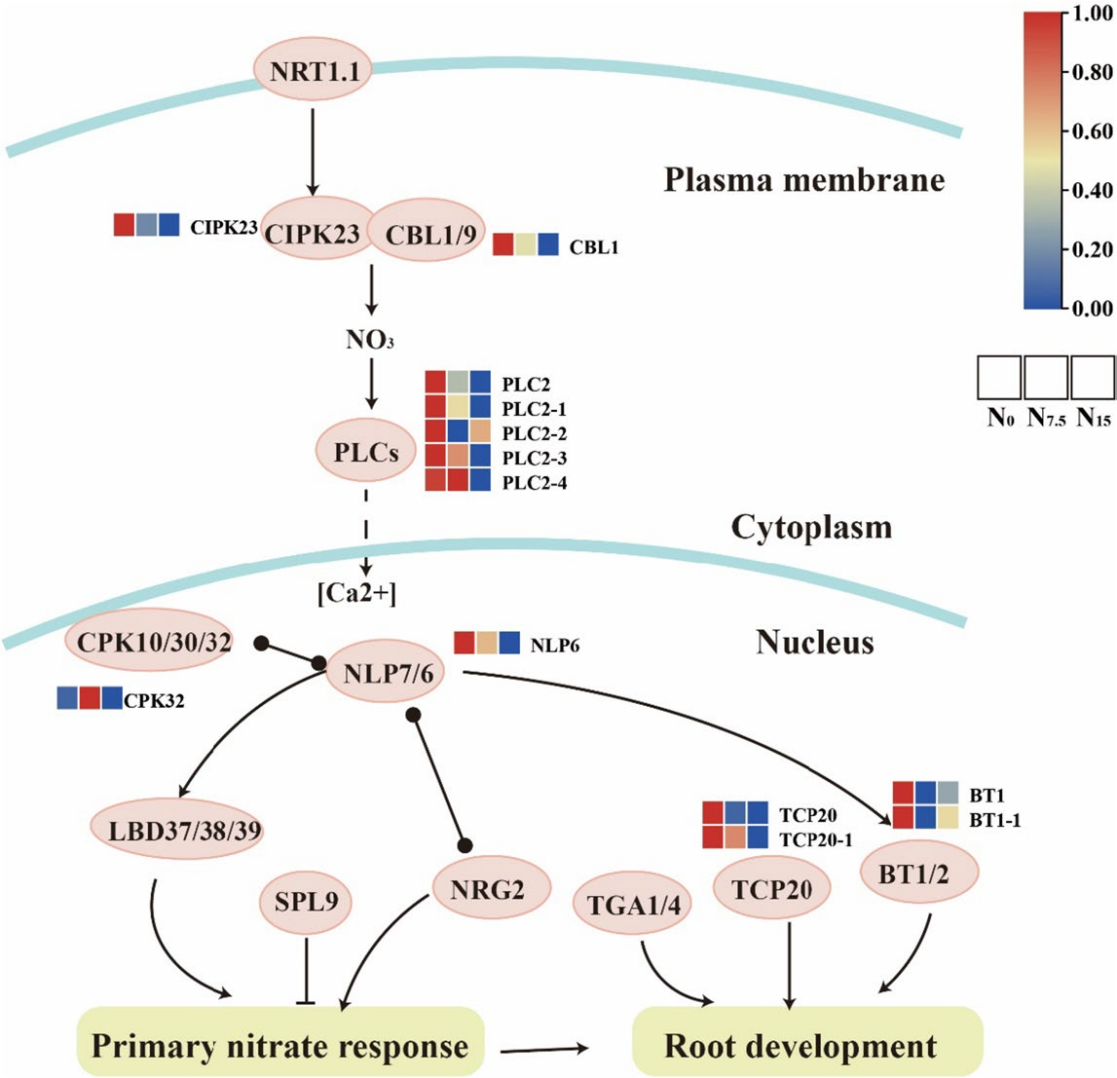
Response of nitrate signal-sensing and transduction pathway-related proteins to N levels. The pathway map was prepared by using KEGG PATHWAY Database. With each box, each column is different nitrogen treatment (from left to right: N_0_, N_7.5_ and N_15_) as shown in the middle upper part. Average proteins intensity is color key scale according the scale in the middle upper part

The expression of genes *CBL9, CPK32, LBD37, LBD38, LBD32, NIA, NIA1* and *SPL9* were significantly up-regulated in the N_7.5_-grown plants (Fig. 5). The expression of NO_3_^-^ signal-sensing and transduction genes were down-regulated in the N_0_-grown plants compared with the N_7.5_- and N_15_-grown plants, including *BT1, CBL1, CIPK23, CPK10, CPK30, NLP6, NLP7, NRG2, PLC2, PLC4, PLC6, TCP20,* and *TGA1*, (Fig. 5). Meanwhile, the expression of proteins CIBK23, CBL1, PCL2, NLP6, BT1, and TCP20 were up-regulated in the N_0_-grown plants compared with the N_7.5_- and N_15_-grown plants (Fig. 6). 6 genes were selected for qRT-PCR verification. The expression trends of NO_3_^-^ signal-sensing and transduction-related genes were consistent with the results of root RNA-seq data (Table S1, Figure S10).

### Combinatorial analysis of proteins and genes

**Fig. 7:**
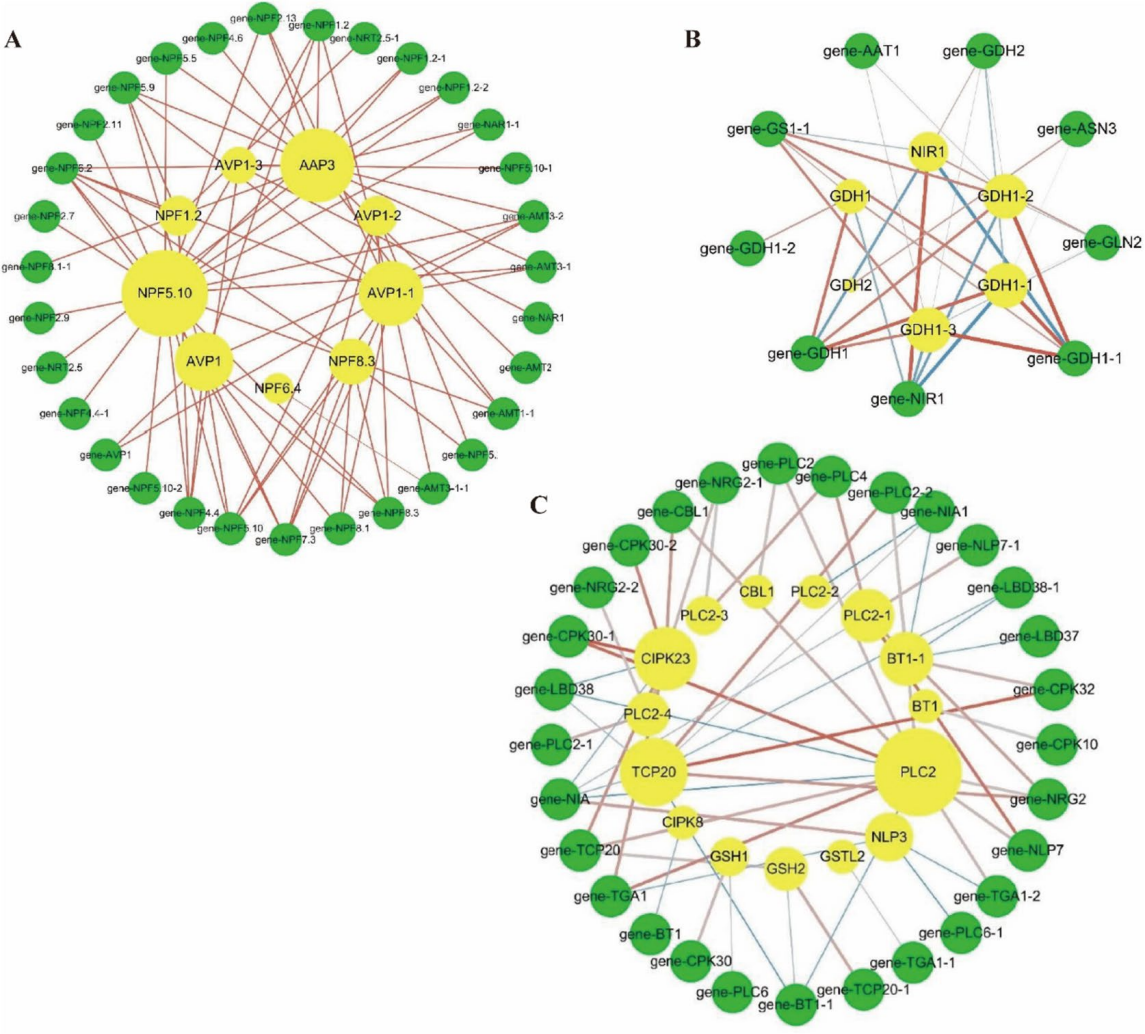
Co-expression analysis of key genes and proteins in the N uptake and transport pathway (**A**). Co-expression analysis of key genes and proteins in the N assimilation pathway (**B**). Co-expression analysis of key genes and proteins in the nitrate signal-sensing and transduction pathway (**C**). Yellow nodes represent proteins (larger sizes are associated with more genes), and green nodes indicates genes. Red edges represent positive correlations (PPC > 0.8) and blue edges represent negative correlations (correlations are stronger with thicker lines)

A network analysis of 31 genes and 9 proteins involved in N uptake and transport pathways was performed (Fig. 7A). Protein NPF5.10 (Pno04G001253), AVP1-1 (Pno02G000314), AVP1 (Pno01G001260), and AAP3 (Pno09G000064) were identified as interact with more than 10 genes (Fig. 7A). Network analysis of 9 genes and 6 proteins involved in N assimilation pathway was performed (Fig. 7B). Protein NIR1 (Pno07G004791) had a strong negative correlation with genes *GDH1 (Pno05G000521)* and *GDH1-1 (Pno877G000001)* (Fig. 7B). There was a strong positive correlation between protein GDH1 and gene *GDH1* (Fig. 7B). 29 genes and 15 proteins were selected from the NO_3_^-^ signal-sensing and transduction pathway for network analysis (Fig. 7C). There was a strong positive correlation between protein TCP20 and gene *CPK32 (Pno08G003704)*. *Gene CPK30-1 (Pno04G005805)* was positively correlated with protein CIPK23 and PLC20 (Fig. 7C).

### 3D structure of the GS1 and GDH1 and their interaction with NH_4_^+^

**Fig. 8:**
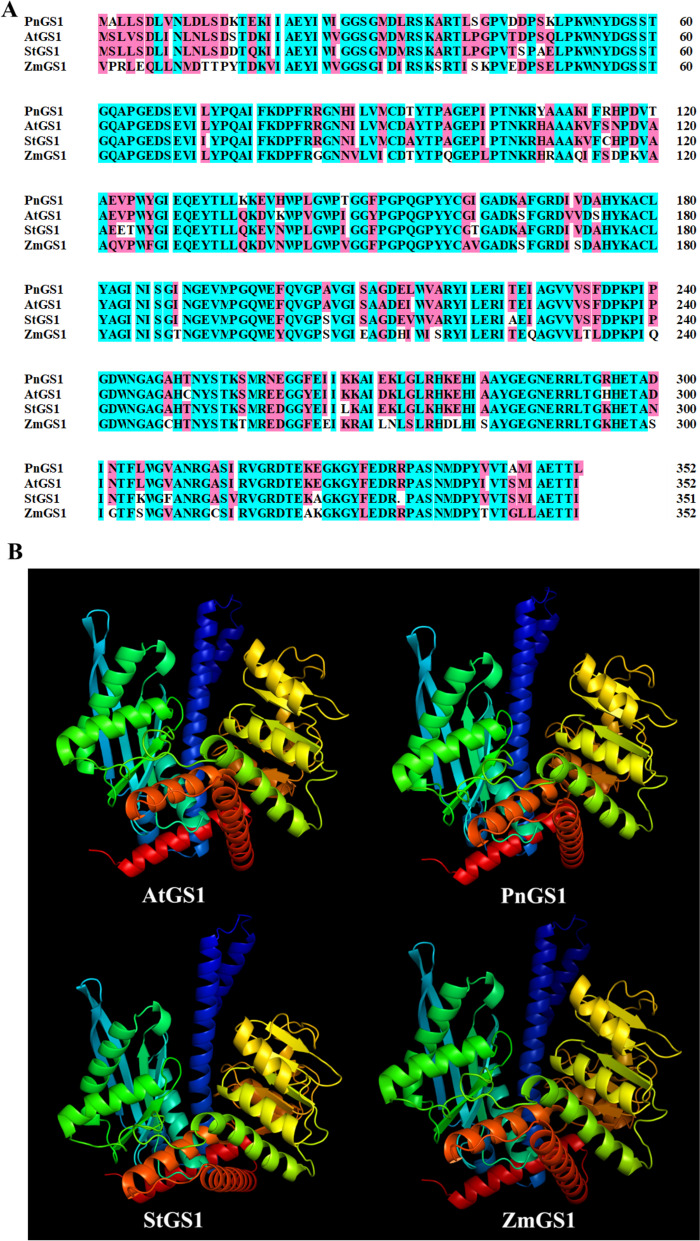
Amino acid sequence alignment and three-dimensional (3D) protein structure prediction of GS1 in *Arabidopsis thaliana* (AtGS1), *P. notoginseng* (PnGS1), *Solanum tuberosum* (StGS1) and *Zea mays* (ZmGS1). The *AtGS1*, *PnGS1*, *StGS1* and *AtGS1* nucleotide sequence was translated into protein sequence (**A**). Highly accurate protein structure prediction with AlphaFold2, save its PDB file and visualize it using PyMOL software, and the predicted 3D protein colored light purple is shown in cartoon representation (**B**)

**Fig. 9:**
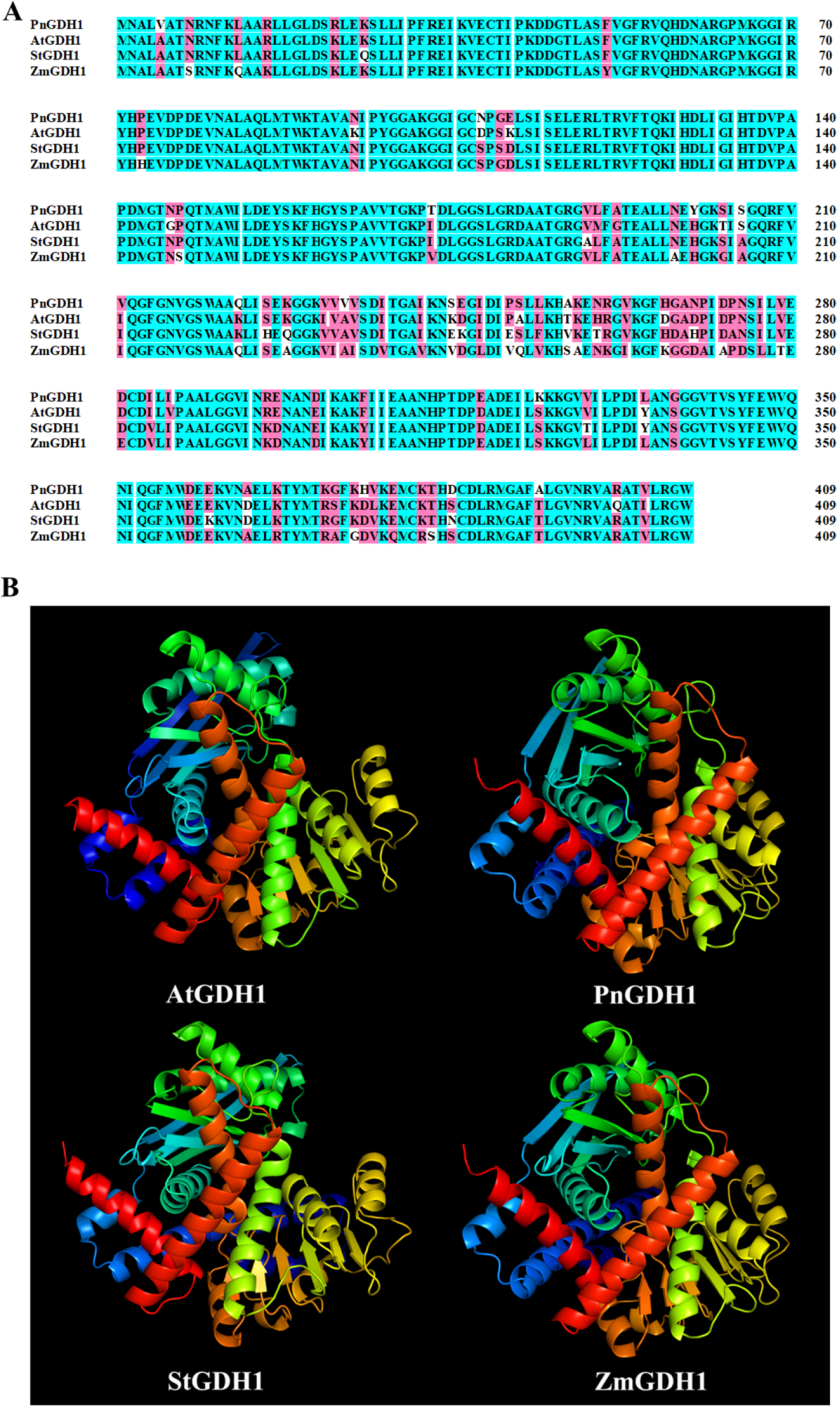
Amino acid sequence alignment and 3D protein structure prediction of GDH1 in in *A*. *thaliana* (AtGDH1), *P*. *notoginseng* (PnGDH1), *S*. *tuberosum* (StGDH1) and *Z*. *mays* (ZmGDH1). The AtGDH1, PnGDH1, StGDH1 and AtGDH1 nucleotide sequence was translated into protein sequence (**A**). Highly accurate protein structure prediction with AlphaFold2, save its PDB file and visualize it using PyMOL software, and the predicted 3D protein colored light purple is shown in cartoon representation (**B**)

**Fig. 10:**
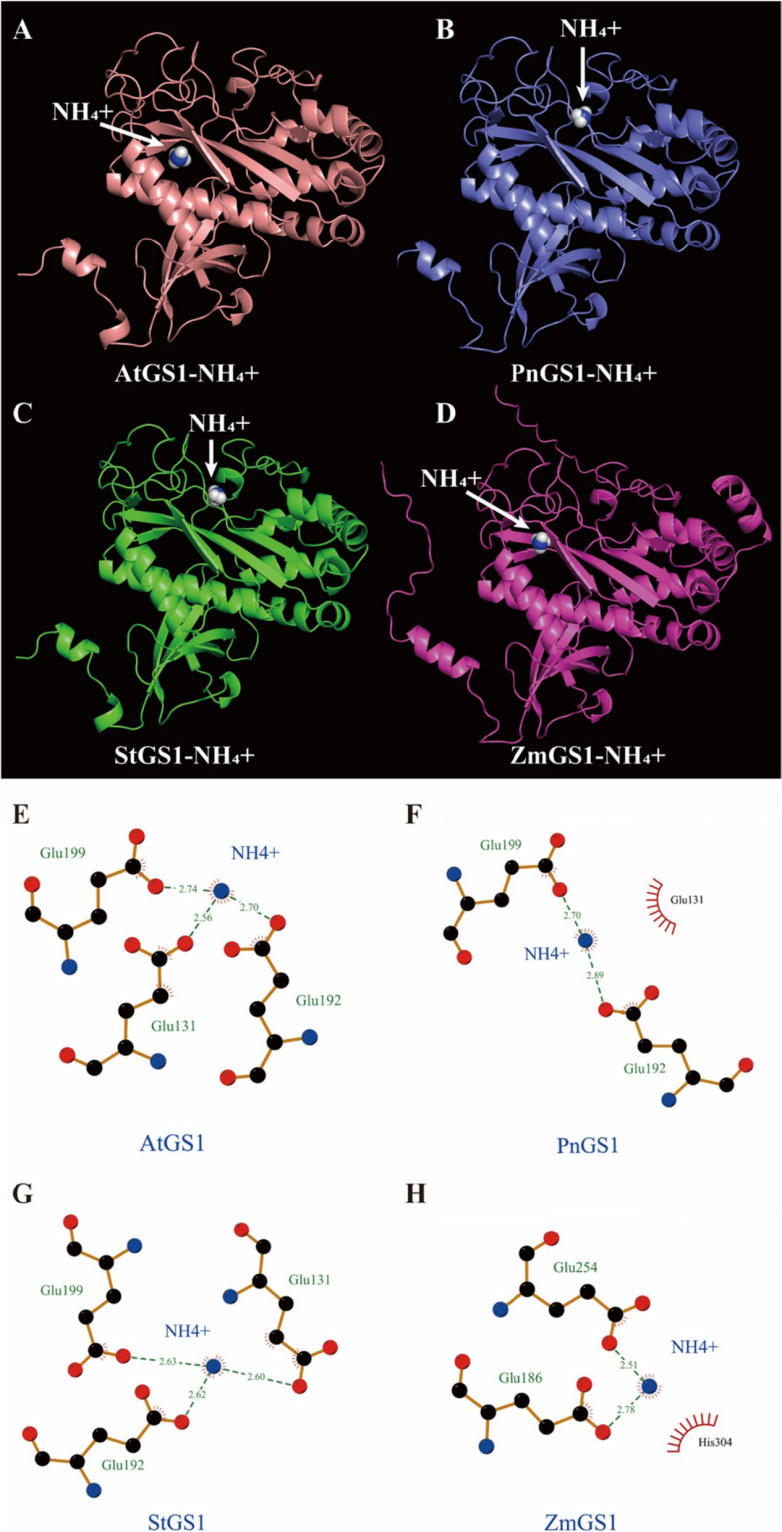
Binding model of GS1 to NH_4_^+^ by molecular docking. The 3D AtGS1-NH_4_^+^ interaction model (**A**). The 3D PnGS1-NH_4_^+^ interaction model (**B**). The 3D StGS1-NH_4_^+^ interaction model (**C**). The 3D ZmGS1-NH_4_^+^ interaction model (**D**). The 2D AtGS1-NH_4_^+^ interaction model (**E**). The 2D PnGS1-NH_4_^+^ interaction model (**F**). The 2D StGS1-NH_4_^+^ interaction model (**G**). The 2D ZmGS1-NH_4_^+^ interaction model (**H**). Dashed lines indicate a potential interaction between amino acid residues with NH_4_^+^. The black balls showed carbon atoms, the blue balls showed nitrogen atoms whereas the red balls showed the oxygen atoms. The residues involved in non-bonding interactions were shown as red bristles. The NH_4_^+^ had hydrophobic interactions with Glu131 and His304

**Fig. 11:**
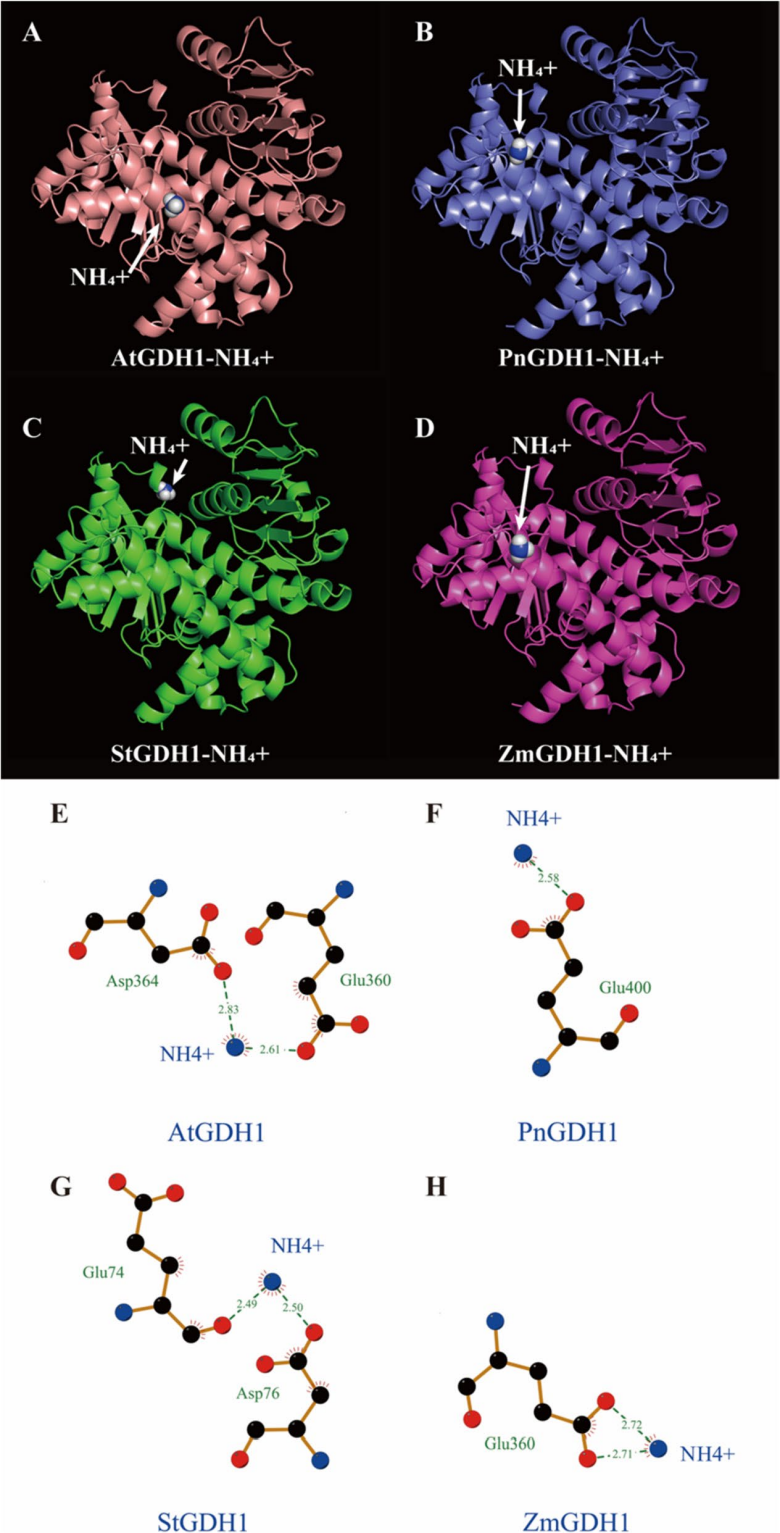
Binding model of GDH1 to NH_4_^+^ by molecular docking. The 3D AtGDH1-NH_4_^+^ interaction model (**A**). The 3D PnGDH1-NH_4_^+^ interaction model (**B**). The 3D StGDH1-NH_4_^+^ interaction model (**C**). The 3D ZmGDH1-NH_4_^+^ interaction model (**D**). The 2D AtGDH1-NH_4_^+^ interaction model (**E**). The 2D PnGDH1-NH_4_^+^ interaction model (**F**). The 2D StGDH1-NH_4_^+^ interaction model (**G**). The 2D ZmGDH1-NH_4_^+^ interaction model (**H**). Dashed lines indicate a potential interaction between amino acid residues with NH_4_^+^. The black balls showed carbon atoms, the blue balls showed nitrogen atoms whereas the red balls showed the oxygen atoms. The residues involved in non-bonding interactions were shown as red bristles

To identify the key amino acid residues in GS1 and GDH1 that affect the NUE and contribute to differences in enzyme activity in *Z. mays*, *A. thaliana*, *Solanum tuberosum* and *P. notoginseng* (Figs. 8B and 9B). 94.10%-99.02% of amino acids of GS1 and GDH1 were located in region where backbone dihedral angles were energetically favored, indicating that this predicted model of GS1 and GDH1 were high quality (Figure S11). As NH_4_^+^ is the major substrate of GS1 and GDH1, we evaluate the affinity of the GS1 and GDH1 with NH_4_^+^ by molecular docking (Figs. 10A-D; 11A-D). The docking sites of PnGS1 and StGS1 with NH_4_^+^ were similar (Figs. 10B, C; S12D). A total of 5 candidate amino acid residues (Glu199, Glu131, Glu192, Glu254 and Glu186) possibly involved in the reaction between NH_4_^+^ and AtGS1/StGS1/PnGS1/ZmGS1 were identified (Figs. 10E-H, S13; Table S2). The 2D PnGS1-NH_4_^+^ interaction analysis revealed that Glu192 and Glu199 directly binding to NH_4_^+^ (Fig. 10F). The docking sites of PnGDH1 and ZmGDH1 with NH_4_^+^ were comparable (Figs. 11B, D; S14B, D). A total of 6 candidate amino acid residues (Glu360, Asp364, Glu400, Glu74, Asp76 and Glu360) possibly involved in the reaction between NH_4_^+^ and AtGDH1/StGDH1/PnGDH1/ZmGDH1 were identified (Fig. 11E-H, Table S3). The 2D PnGDH1-NH_4_^+^ interaction analysis disclosed that Glu400 directly binding to NH_4_^+^ (Fig. 11F).

## Discussion

### N deficiency reduces the occupation of N in root

An enhancement of N uptake efficiency and N use efficiency is a primary strategy to improve N efficiency [25]. The ability of plants to absorb N would be obviously weakened under the low N condition [26]. N uptake considerably affects N use efficiency [27]. Herein, N deficiency enhances NUE, NUEb and RNF (Fig. 1A, C; Table 2), and the proportion of N distribution to root was decreased. These results further confirm that the higher NUE under the low N might be closely linked to the occupation of N by root [28]. The RNL is commonly used to reflect the occupation of N by roots [29]. Our results demonstrated that N deficiency significantly reduces the occupation of N in roots (Fig. 1). It is worth noting that the proportion of N distribution to shoot was increased (Fig. 1B). We speculate that the limited N absorbed by the roots might be more extensively transported to the above-ground parts to maintain the survival under the N-deficient condition [26]. Overall, N deficiency reduces the occupation of N in root and thus changes NUE.

### Low N promotes the efficiency of N uptake and transportation

The uptake of NO_3_^-^ is mainly mediated by NO_3_^-^ transport proteins (NRT/NFP) [9, 30]. NPF8.1, NPF4.6, and NRT2 family (the latter referring to *NRT2.1*, *NRT2.2*, *NRT2.4*, and *NRT2.5*) promote the uptake of NO_3_^-^ in *A. thaliana* and *Panicum miliaceum* grown under the N-deficient condition [31, 32]. N deficiency enhances the expression of NO_3_^-^ transporters genes (*NPF8.1*, *NRT2.5*, *NRT2.1* and *NRT2.2*) (Fig. 2). It has been recorded that *AtAVP1* over-expression has been demonstrated to enhance the expression of *NRT2.1* and biomass accumulation in *Romaine lettuce* [33]. NO_3_^-^ uptake-related genes (as referred to *AVP1*, *NAR1*, *NPF2.11*, *NPF2.13*, *NPF4.3*, *NPF4.5*, and *NFP5*) were up-regulated in the N-deficient plants (Fig. 2). Interestingly, a significant positive correlation between gene *NRT2.5* and protein NPF5.10 and AVP1 was observed (Fig. 7). These results suggest that the uptake of NO_3_^-^ is closely related to the increased expression levels of NO_3_^-^ uptake-related genes and proteins under the N-deficient condition. Furthermore, the uptake of NH_4_^+^ is mainly mediated by NH_4_^+^ transport proteins AMT [9]. The up-regulation expression of *OsAMT1.1* and *OsAMT1.2* considerably increase NH_4_^+^ uptake and significantly improve NUE in the N-deficient *O. sativa* [34, 35]. In the present study, N deficiency increases the uptake of NH_4_^+^ by up-regulating AMT (Figs. 2 and 4). Furthermore, N deficiency up-regulated the expression of gene *NPF1.2* related to NO_3_^-^ transport from roots to shoots, but protein NPF1.2 expression was down-regulated. (Figs. 2 and 4) [36, 37]. In response to the inconsistency between mRNA and protein level, we speculate that *NPF1.2* may have different post-translational modification states before and after N application [38]. In summary, *NPF8.1*, *NPF4.6*, *AMT*, *AVP* and NRT2 family genes play an important role in improving N uptake and transportation under the N-deficient condition.

### The roles of Glu192, Glu199 and Glu400 in the N assimilation

N assimilation is a core process for N utilization in plants [39]. NUE can be enhanced by the expression of N assimilation-related gene *OsNiR* in the N-deficient *O. sativa* [40]. NiR enzyme activity was not significantly different among N regimes (Fig. 1D), but the expression of gene *NIR1* and protein NIR1 up-regulated with increasing N supply (Figs. 3, 4 and 7B). Our results revealed that N supply might promote an increase in NiR quantity, but the structure and composition of NiR did not cause a change in the activity of nitrite (NO_2_^-^) being reduced to NH_4_^+^ under N regimes [27, 41]. Additionally, *GS1-1* is the dominant gene induced by low N to synthesis NH_4_^+^ and glutamine into glutamate [42]. *OsGS1;2* and *DvGS1/2* over-expression has been shown to enhance the total protein content, NUE and biomass accumulation in the N-deficient plants [12–14]. We speculate that the expression of *GS1-1* contributes to the synthesis of glutamate in the N-deficient plants as demonstrated by a number of investigation (Fig. 3) [43]. The expression of gene *ASN3* and protein ASN3 were up-regulated in the N-deficient *P. notoginseng* and *Lactuca sativa*, indicating that more NH_4_^+^ is synthesized into amino acids (Figs. 3 and 4) [44]. A number of studies has demonstrated that the activity of GDH enzyme is increased under the low N condition [45–47]. In the present study, N deficiency up-regulates the expression and activity GDH, and there was a significantly positive correlation between protein GDH1 and gene *GDH1* (Fig. 7B). N mediate post-translational phosphorylation modifications of GDH2, resulting in an inconsistent change at the gene and protein levels (Figs. 3 and 4) [48]. These results indicate that GDH1 might play an important role in response to N deficiency. As such, gene *GS1-1* and protein *GDH1* are suggested as the prospective key genes regulating the N deficiency-driven enhancement of N assimilation, and they contribute to the improvement of NUE under the low N condition.

The method of molecular docking is popular to study the biological activity of N affecting plant NUE [21]. PnGS1-NH_4_^+^ interaction analysis revealed that Glu192 and Glu199 were strictly conserved and located around the middle of the catalytic pocket (Fig. 10). Similar results have been reported in *A. thaliana*, *Z. mays* and *Medicago truncatula* GS proteins [49]. The strictly conserved amino acids Glu192 and Glu199 of GS in *M. truncatula* are positioned approximately in the middle of cavity, favorably located to mediate cation coordination [50, 51]. The gene expression of *GS1* were up-regulated in the N_0_-grown plants (Fig. 3). We speculate that N deficiency induces the conserved residues Glu199 and Glu192 to bind more NH_4_^+^, consequently catalysing the synthesis of more glutamine from glutamate. Additionally, site-directed mutagenesis and 3D structure determination of glucoamylase has identified Glu400 as the general base catalyst, which is conducive to the synthesis of more amino acids [52, 53]. PnGDH1-NH_4_^+^ interaction analysis revealed that Glu400 directly binds to NH_4_^+^ (Fig. 11), and the expression and activity of GDH1 were up-regulated in the N_0_-treated plants (Figs. 1, 3 and 4). These results suggest that the high expression of GDH1 enhances the docking ability of Glu400 to NH_4_^+^ and promotes the synthesis of glutamate in the N-deficient plants. Similarly, the residues of conserved proline and T101 significantly enhance NO_3_^-^ transport activity and NUE in the N-deficient *A. thaliana* [20]. Therefore, N deficiency induces Glu residues (as referred to Glu192, Glu199 and Glu400) to enhance the biological activity of GS1 and GDH1, thereby improving N assimilation. A review of previous studies on usea-urease docking complexes has suggested that different species (as reflected by *Glycine max* and *M. truncatula*) could share common interacting residues as well as may have some other uncommon residues at species-dependent way [54]. In the present study, Glu192 and Glu199 in *P. notoginseng* and *S. tuberosum* were identified as hotspot residues residing in GS1 (Figs. 10, S12). We speculate that the preference for NO_3_^-^ is the main reason for the similar GS1 activity in the rhizomatous species *P. notoginseng* and *S. tuberosum*. Overall, the residues of Glu192, Glu199 and Glu400 are suggested as the prospective key hotspot residues regulating the N deficiency-driven enhancement of N assimilation in the rhizomatous species.

### N deficiency induces NO_3_^-^ signal-sensing and transduction

NO_3_^-^ would regulate plant N uptake through signal transduction, thereby changing NUE [55]. The NO_3_^-^ signal-sensing and transduction is regulated by the interaction of NRT1.1 and CIPK, in which gene *CIPK23* specifically promotes NRT1.1 phosphorylation under the N deficiency condition, thus promoting high-affinity response [20]. N deficiency induces the sensing of NO_3_^-^ signal by up-regulating gene *CIPK23* and protein *CIPK23* expression (Figs. 5 and 6). NO_3_^-^ signals trigger downstream NO_3_^-^ responses through calcium (Ca^2+^) -dependent and Ca^2+^-independent pathways, and the accumulation of Ca^2+^ requires the accumulation of phospholipase C (PLC) [55]. The upregulation of PLC-related genes and proteins in this study supports the view that N deficiency promotes the transduction of NO_3_^-^ signal (Figs. 5 and 6, S10). Meanwhile, NLP6/7 (the main regulatory factor for primary NO_3_^-^ response) and numerous transcription factors (*LBD37/38/39*, *NRG2*, *TGA1/4*, *TCP20*, and *BT1/2*) positively regulate the primary NO_3_^-^ responses under the N-deficient condition [16, 55–57]. Proteins NLP6, TCP20, BT1, and genes *NLP6*, *NLP7*, *LBD37*, *NRG2*, *TCP20*, *BT1* were generally up-regulated in the N_0_-grown plants (Figs. 5 and 6). These results indicate that the NO_3_^-^ signal-sensing and transduction are enhanced in *P. notoginseng* under the N-deficient condition.

## Conclusion

**Fig. 12:**
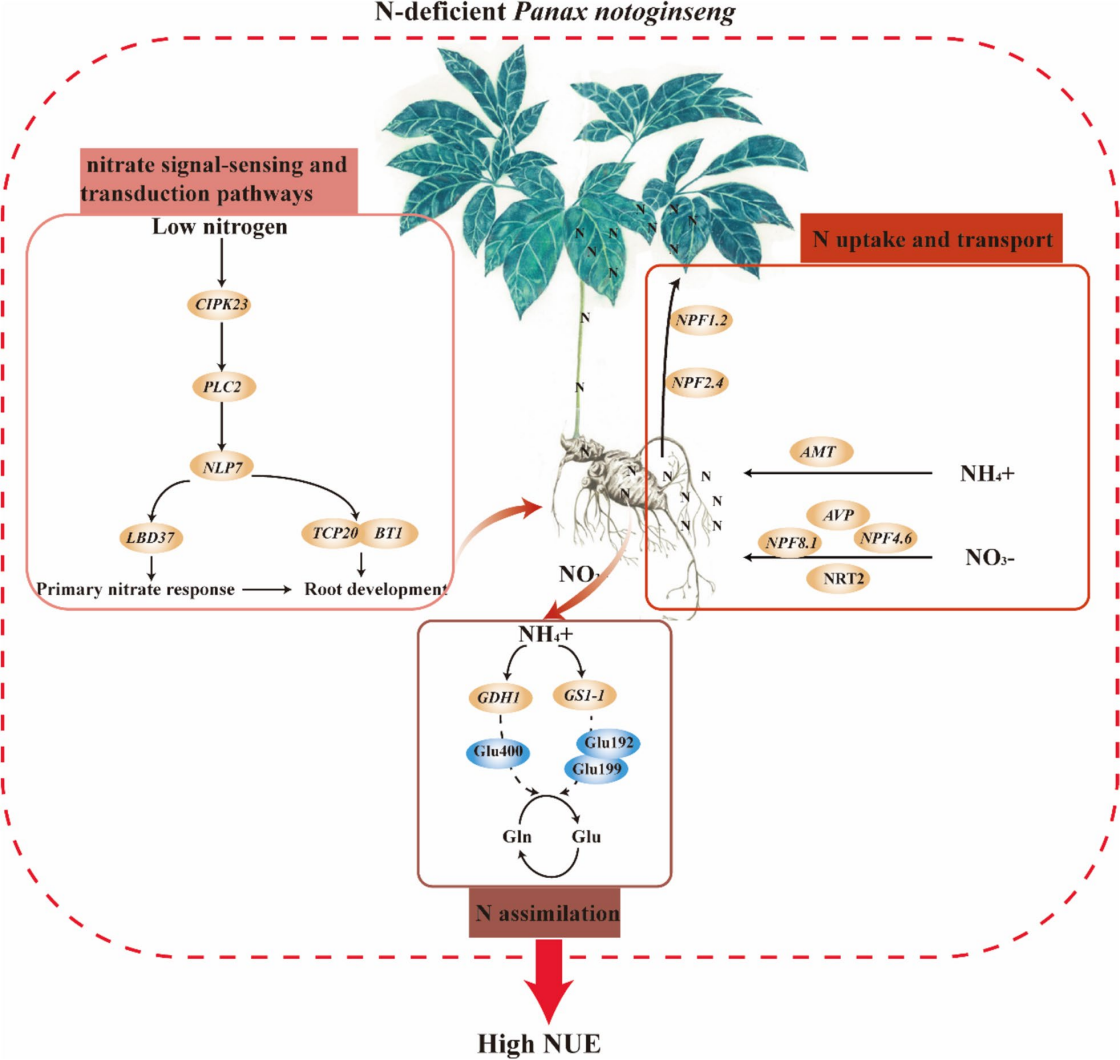
A regulatory mechanism of N deficiency-driven enhancement of NUE has been proposed in the rhizomatous species *P. notoginseng*

We have proposed a hypothetical genetic regulatory network (Fig. 12), and have suggested that N deficiency promotes the expression of key genes involved in N uptake, transport, assimilation, signaling and transduction, and thus enhance NUE in the rhizomatous medicinal plant *P. notoginseng*. *NPF8.1*, *NPF4.6*, *AMT*, *AVP* and NRT2 family genes might be considered as the key genes regulating the N deficiency-promoted N uptake. The genes of *NPF1.2* and *NRT2.4* might mediate N deficiency-induced N transport from roots to shoots. N deficiency would induce the residues of Glu192, Glu199 and Glu400 to enhance the biological activity and expression of GS1 and GDH1, thereby improving N assimilation. The expression of genes *CIPK23*, *PLC2*, *NLP6*, *TCP20*, and *BT1*, contributes to NO_3_^-^ signal-sensing and transduction under the N-deficient condition. In summary, the genes and residues proposed as being involved in N metabolism would provide excellent candidates for further genetic improvement in the NUE of rhizomatous medicinal plants.

## Materials and methods

### Experiment design

A potted experiment was conducted from January to November 2021 in Kunming, Yunnan Province, southwestern China (longitude 102°45', latitude 25°08'). Seedlings were harvested from the plants of 1-year-old *P. notoginseng* (Burk.) F. H. Chen. that were cultivated at the experimental farm of Wenshan Miao Xiang *P. notoginseng* Industrial Co., Ltd., China. Healthy rhizome of *P. notoginseng* were selected in our experiments and transplanted to a plastic pot (35×40 cm) on January 2021. The pot experiment was conducted in a controlled environment growth chamber with a light intensity of about 10% [58]. The physicochemical properties of the soil are shown as following: pH 6.02, organic matter 11.04 g·kg^-1^, hydrolysable N 54.58 mg·kg^-1^, available phosphorus (P) 0.46 mg·kg^-1^, available potassium (K) 36.67 mg·kg^-1^, total N 0.10%, total phosphorus 0.10%, total potassium 0.79 g·kg^-1^.

In the present study, a completely randomized design was used with three replicates for each treatment, including three N addition levels (Figure S1): (i) without N addition (without N addition), N_0_; (ii) 112.5 kg·N·ha^-1^ (mild N deficiency), N_7.5_; (iii) 225 kg·N·ha^-1^ (normal N), N_15_. Each replicate consisted of 40 pots. In addition to N fertilizer, the application levels of phosphorus fertilizer (225 kg·P_2_O_5_·ha^-1^) and potassium fertilizer (450 kg·K_2_O·ha^-1^) were the same for all treatments. The fertilizers applied in the study were compound fertilizer (N:P:K = 32:4:0), calcium superphosphate (N:P:K = 0:52:34), and potassium sulfate (N:P:K = 0:0:52). Fertilization was applied four times in mid-May, June, July, and mid-August 2021. Conventional pesticides were used to control weeds, diseases, and pests. In November 2021, roots were collected under different N treatments, washed, and then flash-frozen in liquid N and stored at -80°C for transcriptome and metabolome analyse.

### Determination of C and N content

Plant was separated into taproot, rhizome, fibrous roots, stem and leaf. The taproot, rhizome, fibrous roots, stem and leaf were dried at 60℃ to constant weight for 96 h. Dry matter was determined. The dried samples were ground and passed through a 100-mesh sieve for further analysis. The C and N content were determined using an elemental analyzer (Vario EL III; Elementar analysisysteme GmbH Hanau, Germany).

### Calculation of N use efficiency

Based on biomass and N content, N uptake and utilization related parameters were calculated as described by Gupta et al. [59]: Total N uptake (TN, mg·plant^-1^) = above-ground N content + below-ground N content; Total N uptake per unit root length (TNL, mg·cm^-1^) = total N uptake per plant / total root length per plant; Root N content per unit root length (RNL, mg·cm^-1^) = Root N content / total root length per plant; Proportion of N distribution to shoot (%) = above-ground N content / total N uptake; Proportion of N distribution to root (%) = root N content / total N uptake; N use efficiency (NUE, kg·kg^-1^) = yield (below-ground dry weight) / total N uptake; N agronomic efficiency (NAE, kg·kg^-1^) = (yield with N application – yield without N application) / N application rate; Recovery of N fertilizer (RNF, %) = (above-ground N content with N application – above-ground N content without N application)/N application rate × 100; N contribution rate (NCR, %) = (yield with N application – yield without N application) / yield with N application × 100; N partial factor productivity (NPFP, kg·kg^-1^) = yield with N application / N application rate; N use efficiency in biomass production (NUEb, g·DW·g^-1^·N) = below-ground dry matter accumulation per plant / below-ground N accumulation per plant; Harvest index = root dry weight at harvest / plant dry weight at harvest; N harvest index = N uptake in roots at harvest / total N accumulation per plant; N uptake efficiency (kg·kg^-1^) = total N uptake per plant / N application rate.

### Determination of enzyme activity

The activity of N metabolism-related enzymes was assayed according to Li [60]. The measurement of the activity of NiR (G0408F), NADH-GOGAT (G0403F), GS (G0401F), and NADH-GDH (G0405F) were carried out by kits from Suzhou Gores Biotechnology Co., Ltd.

### RNA-Seq and annotation

The experimental process of transcriptome sequencing includes RNA extraction, RNA quality detection, library construction, and sequencing. Firstly, total RNA was extracted from the roots of *P. notoginseng*. The integrity of the sample RNA and the presence of DNA contamination were examined using an RNase-free agarose gel electrophoresis. Then, enriched mRNA fragments were fragmented into short fragments using a fragmentation buffer, and reverse transcribed into cDNA using Illumina's NEBNext Ultra RNA Library Prep Kit (NEB#7530, New England Biolabs, Ipswich, MA, USA). The purified double-stranded cDNA was end-repaired, A-tailed, and ligated with sequencing adapters. 200 bp cDNA was selected using AMPure XP beads, PCR amplified, and purified with AMPure XP beads again to obtain the library. Guangzhou Genedenovo Biotechnology Co., Ltd. sequenced the obtained cDNA library on the Illumina Novaseq6000 platform. Finally, 9 cDNA libraries representing three replicates and three N levels were constructed, and the transcriptome was sequenced on the Illumina Hiseq platform using *P. notoginseng* genome (PRJNA608068; http://herbalplant.ynau.edu.cn/) as the reference genome [61]. After obtaining the gene expression levels for each sample, differentially expressed genes (DEGs) among samples were analyzed. The input data for gene differential expression analysis were the reads count data obtained in the gene expression level analysis, and DESeq2 software was used for analysis. Based on the differential analysis results, genes with FDR (false discovery rate) < 0.05 and |log_2_FC| > 1were defined as significantly differentially expressed genes [62, 63]. After selecting differentially expressed genes according to the analysis purpose, a clustering heat map of different samples was generated for functional annotation, enrichment analysis, trend analysis, and other analyses of DEGs.

### Determination and analysis of data-independent acquisition (DIA) proteomics

In November 2021, samples grown under different N treatments were collected and immediately washed, frozen in liquid N, and stored at -80℃ for DIA protein profiling analysis. DIA protein profiling analysis included processes such as protein extraction, denaturation, reduction, alkylation, enzymatic digestion, and desalting of peptides. The tissue samples were pre-processed using the iST sample pre-processing kit (PreOmics, Germany). After grinding the samples in liquid N, an appropriate amount of sample was taken and added to 50 µL of lysis buffer. The mixture was then heated at 95℃ with 1000 rpm for 10 min. After cooling to room temperature, the sample was incubated with trypsin digestion buffer at 37℃ and 500 rpm for 2 h. The reaction was then stopped by adding the stop buffer. Peptide desalting was carried out using the iST cartridge provided in the kit, with 2 × 100 µL wash buffer for elution. The eluted peptides were vacuum-dried and stored at -80℃. For analysis, each sample was mixed with 30 µL of solvent A (A: 0.1% formic acid aqueous solution) to form a suspension, and 9 µL of the mixture was taken and mixed with 1 µL of 10 × iRT peptides. The mixture was then separated using nano-LC and analyzed using online electrospray ionization tandem mass spectrometry. Before mass spectrometry detection, Biognosys iRT Kit was added to each sample as a quality control reagent, and the retention time (RT) of peptides in chromatography was calibrated using the QuiC (Biognosys) [64] software for quality control of the raw mass spectrometry data. The Pulsar [65] software was then used to build a database and analyze the DIA data results based on the DDA reference database to identify proteins. Qualitative results and quantitative results in all samples were output when a protein was detected.

The detected protein group was annotated using the GO, KEGG, and KOG databases [66], and the annotation results were statistically analyzed. Based on the expression results of each sample, PCA analysis was used to analyze and calculate the Pearson correlation coefficient between samples to understand the repeatability of the samples and help exclude outliers. Subsequently, proteins with significant differences between groups were selected based on the absolute value of FC greater than 1.5 (|log_2_(1.5)| ≈ 0.58, *P* < 0.05), and the differentially expressed proteins were subjected to GO, KEGG, GSEA, interaction network, and trend analysis.

### Combined transcriptome and proteome analysis

Differently expressed genes and differentially expressed proteins are analyzed in combination The KEGG pathway was mapped to genes and proteins with changed transcriptomics and proteomics, and histograms were produced to show pathway enrichment with differential proteins and genes. Correlation analysis was performed for DEGs and proteins in each group. Pearson’s correlation coefficients (PCCs) were determined using the Cor tool in R (www.r-project.org). Genes and proteins with a |PPC| > 0.80 were created a network diagram to show correlation.

### Sequence alignment and molecular docking

Full-length amino acid sequences of *Z. mays, A. thaliana* and *S. tuberosum* were downloaded from NCBI database (https://www.ncbi.nlm.nih.gov/). Multiple sequence alignment of GS1 and GDH1 in *P. notoginseng, Z. mays, A. thaliana* and *S. tuberosum*, respectively (Figs 8A and 9A). Multiple sequence alignment was visualized using DNAMAN software. 3D structure of the GS1 and GDH1 in *P. notoginseng, Z. mays, A. thaliana* and *S. tuberosum* were predicted using the AlpaFold2 software [67] following the instructions on the website https://github.com/deepmind/alphafold (Figs 8B and 9B). The protein model of GS1 and GDH1 were docked with NH_4_^+^ by AutoDock4 [68]. All model were visualized by PyMOL (http://www.pymol.org/) and Ligplot 2.2.4 [69]. The molecular docking figures were drawn by ourselves.

### qRT-PCR verification

qRT-PCR was performed according to the method described by Xiong et al. [70]. *YLS8* was used as the reference gene [22]. Primers of qRT-PCR are listed in Table S1. Three replicates were performed for each gene and sample. Results were calculated using the formula 2^-ΔΔCt^.

### Statistical analysis

GraphPad Prism 8 (GraphPad Software Inc. USA) and IBM SPSS Statistics 20.0 (IBM Corp. USA) was used for statistical analyses. One-way analysis of variance (ANOVA) test was performed to compare the differences between N regimes. The integrative analysis was performed using the R software, and the co-expression networks were visualized using the Cytoscape (version 3.7.1).

### Supplementary Information

**Additional file 1: Figure S1. ***Panax notoginseng* pot culture under different nitrogen (N) levels, cited from our research group (Cun et al., 2022). **Figure S2.** Protein (A) and peptide (B) identification. **Figure S3.** Annotated Venn diagrams of GO, KEGG, and KOG databases (A), comparison of differentially expressed proteins among N_0_, N_7.5_ and N_15_ (B). Red represents the up-regulated proteins, green represents the up-regulated proteins. **Figure S4.** GO analysis of differentially expressed proteins from two comparation group. (A) N_0_ vs N_7.5_, (B) N_0_ vs N_15_. Red represents the up-regulated proteins, green represents the down-regulated proteins. **Figure S5.** KEGG enrichment analysis for differentially expressed proteins. (A) N_0_ vs N_7.5_, (B) N_0_ vs N_15_. Red represents the up-regulated proteins, blue represents the down-regulated proteins. **Figure S6.** Cluster of proteins expression patterns in response to N regimes. **Figure S7.** Enrichment of functional categories of each cluster with the significantly enriched KEGG pathways plotted for differentially expressed proteins among N regimes. **Figure S8.** Real-time quantitative polymerase chain reaction (qRT-PCR) validation of key genes involved in N uptake and transport in *P. notoginseng*. The left Y-axis and histogram are candidate genes expression obtained via qRT-PCR, the right Y-axis and red line are gene expression level calculated as FPKM value. The relative expression obtained from real-time PCR calculated by 2^−^^△△Ct^ method. Values for histogram were means ± SD (*n* = 3), and significant differences are indicated by letters (ANOVA; *P* < 0.05). The value of each red dot is the average of three biological replicates (*n* = 3). **Figure S9.** qRT-PCR validation of key genes involved in N assimilation in *P. notoginseng* by RNA-seq. The left Y-axis and histogram are candidate genes expression obtained via qRT-PCR, the right Y-axis and red line are gene expression level calculated as FPKM value. The relative expression obtained from real-time PCR calculated by 2^−^^△△Ct^ method. Values for histogram were means ± SD (*n* = 3), and significant differences are indicated by letters (ANOVA; *P* < 0.05). The value of each red dot is the average of three biological replicates (*n* = 3). **Figure S10.** qRT-PCR validation of key genes involved in nitrate signal-sensing and transduction in *P. notoginseng* by RNA-seq. The left Y-axis and histogram are candidate genes expression obtained via qRT-PCR, the right Y-axis and red line are gene expression level calculated as FPKM value. The relative expression obtained from real-time PCR calculated by 2^−^^△△Ct^ method. Values for histogram were means ± SD (*n* = 3), and significant differences are indicated by letters (ANOVA; *P* < 0.05). The value of each red dot is the average of three biological replicates (*n* = 3). **Figure S11.** The ramachandran plot paragraph of GS1 and GDH1. *Arabidopsis thaliana* (AtGS1, A), *Panax notoginseng* (PnGS1, B), *Solanum tuberosum* (StGS1, C), *Zea mays* (ZmGS1, D), AtGDH1 (E), PnGDH1 (F), StGDH1 (G) and AtGDH1 (H). **Figure S12.** Comparison of GS1-NH_4_^+^ interaction models among different species. PnGS1/AtGS1-NH_4_^+^ (A); PnGS1/StGS1-NH_4_^+^ (B); PnGS1/ZmGS1-NH_4_^+^ (C); PnGS1/AtGS1/StGS1/ZmGS1-NH_4_^+^ (D). The bule indicated PnGS1-NH_4_^+^ interaction models. White arrows represent NH_4_^+^ ligands that bind to PnGS1. **Figure S13.** Protein-ligand interaction plot of NH_4_^+^ bound to AtGS1 (A), PnGS1 (B), StGS1 (C) and ZmGS1 (D). The stick indicated the amino acid residue that interacts with the NH_4_^+^ at a distance of 4Å, and hydrophobic bonds were formed between the amino acid residues and the ligand. **Figure S14.** Comparison of GDH1-NH_4_^+^ interaction models among different species. PnGDH1/AtGDH1-NH_4_^+^ (A); PnGDH1/StGDH1-NH_4_^+^ (B); PnGDH1/ZmGDH1-NH_4_^+^ (C); PnGDH1/AtGDH1/StGDH1/ZmGDH1-NH_4_^+^ (D). The bule indicated PnGDH1-NH_4_^+^ interaction models. White arrows represent NH_4_^+^ ligands that bind to PnGDH1. **Figure S15.** Protein-ligand interaction plot of NH_4_^+^ bound to AtGDH1 (A), PnGDH1 (B), StGDH1 (C) and ZmGDH1 (D). The stick indicated the amino acid residue that interacts with the NH_4_^+^ at a distance of 4Å, and hydrophobic bonds were formed between the amino acid residues and the ligand.

**Additional file 2: Tabel S1.** List of primers used in qRT-PCR analysis.

**Additional file 3: Tabel S2.** Comparison of amino acid residue sites in docking different GS1-NH_4_^+^ models.

**Additional file 4: Tabel S3.** Comparison of amino acid residue sites in docking different GDH1-NH_4_^+^ models.

## Data Availability

All data generated or analysed during this study are included in this published article and its supplementary information files. The raw RNA-Seq data from this study have been deposited into the NCBI BioProject with the accession number PRJNA997624 (https://www.ncbi.nlm.nih.gov/sra/PRJNA997624). The molecular docking figures were drawn by ourselves.

## Abbreviations

3D: Three-dimensional
DEGs: Differentially expressed genes
DIA: Data-independent acquisition
FC: Fold change
FDR: False discovery rate
GDH: Glutamate dehydrogenase
GOGAT: Glutamate synthetase
GS: Glutamine synthetase
HATS: High-affinity transport system
LATS: Low-affinity transport system
N: Nitrogen
NAE: N agronomic efficiency
NCR: N contribution rate
NH_4_^+^: Ammonium
NiR: Nitrite reductase
NO_3_^-^: Nitrate
NPFP: N partial factor productivity
NUE: N use efficiency
NUEb: N use efficiency in biomass production
PCCs: Pearson’s correlation coefficients
PLC: Phospholipase C
qRT-PCR: Real-time quantitative reverse transcription
RNF: Recovery of N fertilizer
RNL: Root N content per unit root length
TN: Total N uptake
TNL: Total N uptake per unit root length
