# Supplementary material for: Identification of candidate genes and residues for improving nitrogen use efficiency in the N-sensitive medicinal plant Panax notoginseng
Source: BMC Plant Biol. 2024 Feb 12;24:105. doi: 10.1186/s12870-024-04768-4 (PMC10860327; doi:10.1186/s12870-024-04768-4)

# LEGEND FOR SUPPLEMENTARY FIGURE

**Figure S1** *Panax notoginseng* pot culture under different nitrogen (N) levels, cited from our research group (Cun et al., 2022).

**Figure S2** Protein (**A**) and peptide (**B**) identification

**Figure S3** Annotated Venn diagrams of GO, KEGG, and KOG databases (**A**), comparison of differentially expressed proteins among N_0_, N_7.5_ and N_15_ (**B**). Red represents the up-regulated proteins, green represents the up-regulated proteins.

**Figure S4** GO analysis of differentially expressed proteins from two comparation group. (**A**) N_0_ vs N_7.5_, (**B**) N_0_ vs N_15_. Red represents the up-regulated proteins, green represents the down-regulated proteins.

**Figure S5** KEGG enrichment analysis for differentially expressed proteins. (**A**) N_0_ vs N_7.5_, (**B**) N_0_ vs N_15_. Red represents the up-regulated proteins, blue represents the down-regulated proteins.

**Figure S6** Cluster of proteins expression patterns in response to N regimes.

**Figure S7** Enrichment of functional categories of each cluster with the significantly enriched KEGG pathways plotted for differentially expressed proteins among N regimes.

**Figure S8** Real-time quantitative polymerase chain reaction (qRT-PCR) validation of key genes involved in N uptake and transport in *P. notoginseng*. The left Y-axis and histogram are candidate genes expression obtained via qRT-PCR, the right Y-axis and red line are gene expression level calculated as FPKM value. The relative expression obtained from real-time PCR calculated by 2^−△△Ct^ method. Values for histogram were means ± SD (*n* = 3), and significant differences are indicated by letters (ANOVA; *P* < 0.05). The value of each red dot is the average of three biological replicates (*n* = 3).

**Figure S9** qRT-PCR validation of key genes involved in N assimilation in *P. notoginseng* by RNA-seq. The left Y-axis and histogram are candidate genes expression obtained via qRT-PCR, the right Y-axis and red line are gene expression level calculated as FPKM value. The relative expression obtained from real-time PCR calculated by 2^−△△Ct^ method. Values for histogram were means ± SD (*n* = 3), and significant differences are indicated by letters (ANOVA; *P* < 0.05). The value of each red dot is the average of three biological replicates (*n* = 3).

**Figure S10** qRT-PCR validation of key genes involved in nitrate signal-sensing and transduction in *P. notoginseng* by RNA-seq. The left Y-axis and histogram are candidate genes expression obtained via qRT-PCR, the right Y-axis and red line are gene expression level calculated as FPKM value. The relative expression obtained from real-time PCR calculated by 2^−△△Ct^ method. Values for histogram were means ± SD (*n* = 3), and significant differences are indicated by letters (ANOVA; *P* < 0.05). The value of each red dot is the average of three biological replicates (*n* = 3).

**Figure S11** The ramachandran plot paragraph of GS1 and GDH1. *Arabidopsis thaliana* (AtGS1, **A**), *Panax notoginseng* (PnGS1, **B**), *Solanum tuberosum* (StGS1, **C**), *Zea mays* (ZmGS1, **D**), AtGDH1 (**E**), PnGDH1 (**F**), StGDH1 (**G**) and AtGDH1 (**H**).

**Figure S12** Comparison of GS1-NH_4_^+^ interaction models among different species. PnGS1/AtGS1-NH_4_^+^ (**A**); PnGS1/StGS1-NH_4_^+^ (**B**); PnGS1/ZmGS1-NH_4_^+^ (**C**); PnGS1/AtGS1/StGS1/ZmGS1-NH_4_^+^ (**D**). The bule indicated PnGS1-NH_4_^+^ interaction models. White arrows represent NH_4_^+^ ligands that bind to PnGS1.

**Figure S13** Protein-ligand interaction plot of NH_4_^+^ bound to AtGS1 (**A**), PnGS1 (**B**), StGS1 (**C**) and ZmGS1 (**D**). The stick indicated the amino acid residue that interacts with the NH_4_^+^ at a distance of 4Å, and hydrophobic bonds were formed between the amino acid residues and the ligand.

**Figure S14** Comparison of GDH1-NH_4_^+^ interaction models among different species. PnGDH1/AtGDH1-NH_4_^+^ (**A**); PnGDH1/StGDH1-NH_4_^+^ (**B**); PnGDH1/ZmGDH1-NH_4_^+^ (**C**); PnGDH1/AtGDH1/StGDH1/ZmGDH1-NH_4_^+^ (**D**). The bule indicated PnGDH1-NH_4_^+^ interaction models. White arrows represent NH_4_^+^ ligands that bind to PnGDH1.

**Figure S15** Protein-ligand interaction plot of NH_4_^+^ bound to AtGDH1 (**A**), PnGDH1 (**B**), StGDH1 (**C**) and ZmGDH1 (**D**). The stick indicated the amino acid residue that interacts with the NH_4_^+^ at a distance of 4Å, and hydrophobic bonds were formed between the amino acid residues and the ligand.

**Figure S1**


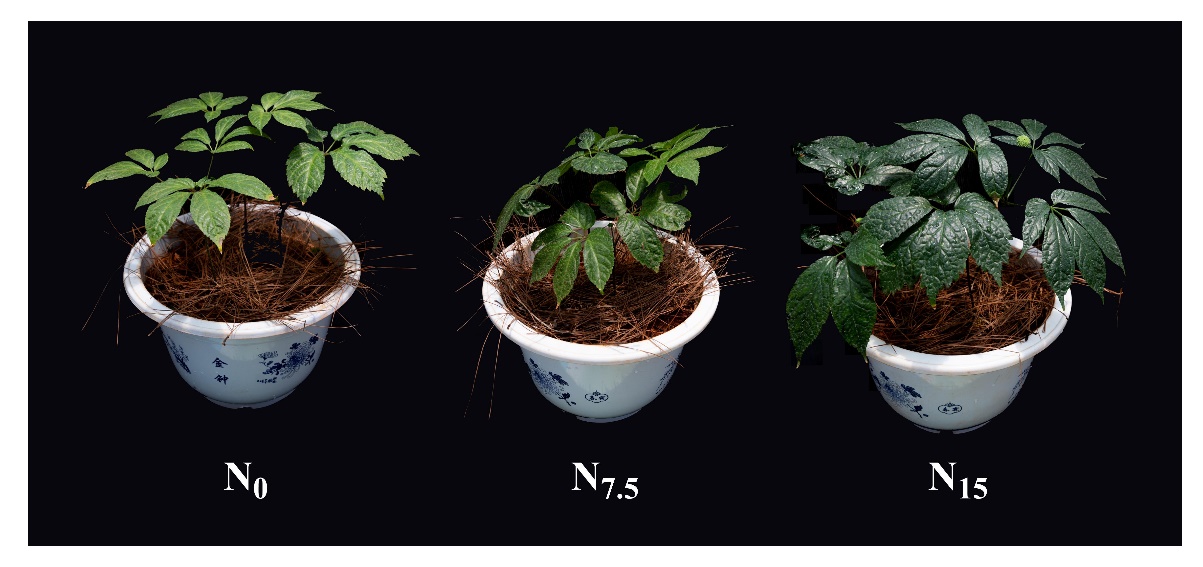


**Figure S2**


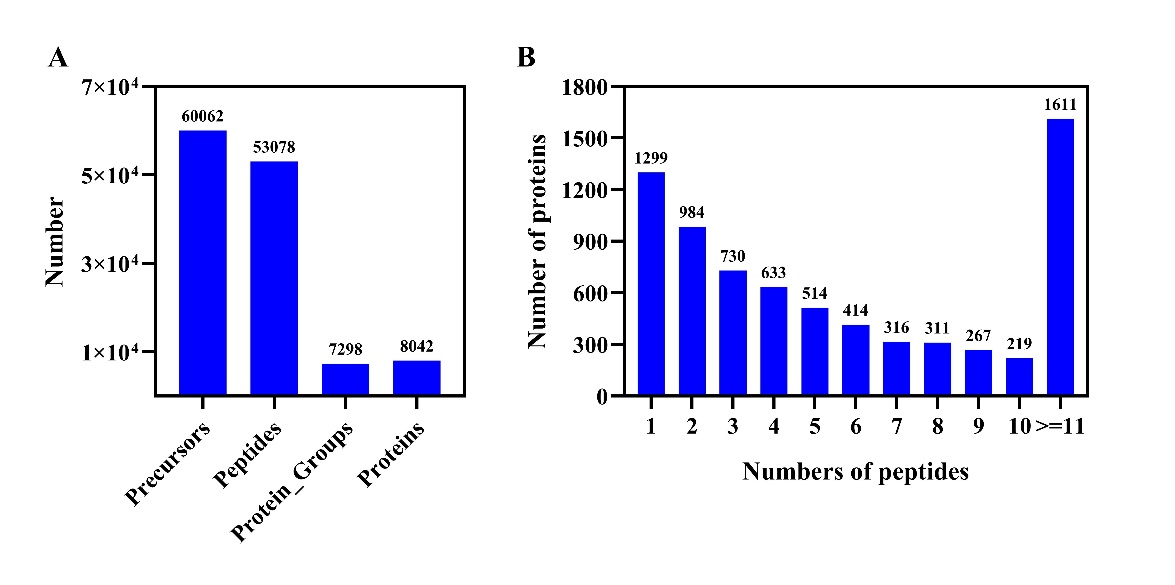


**Figure S3**


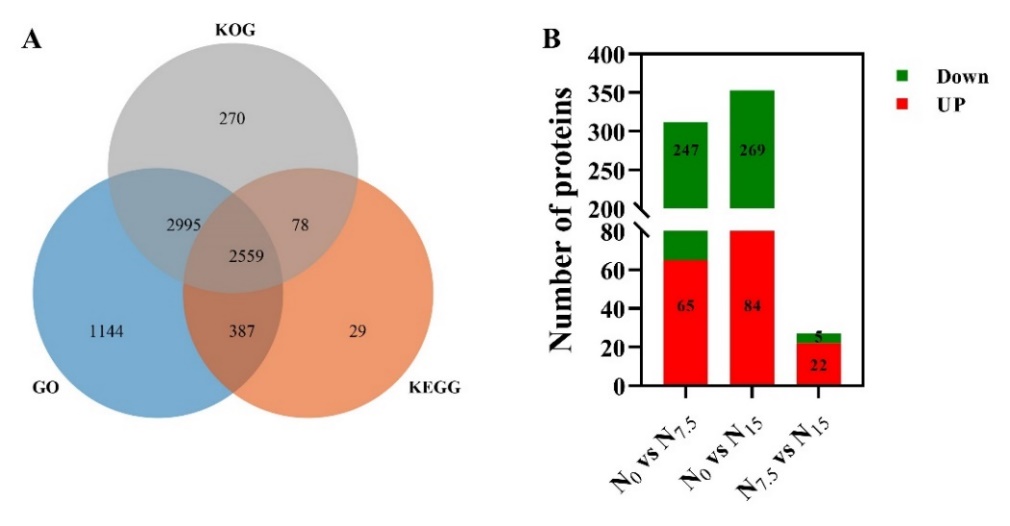


**Figure S4**


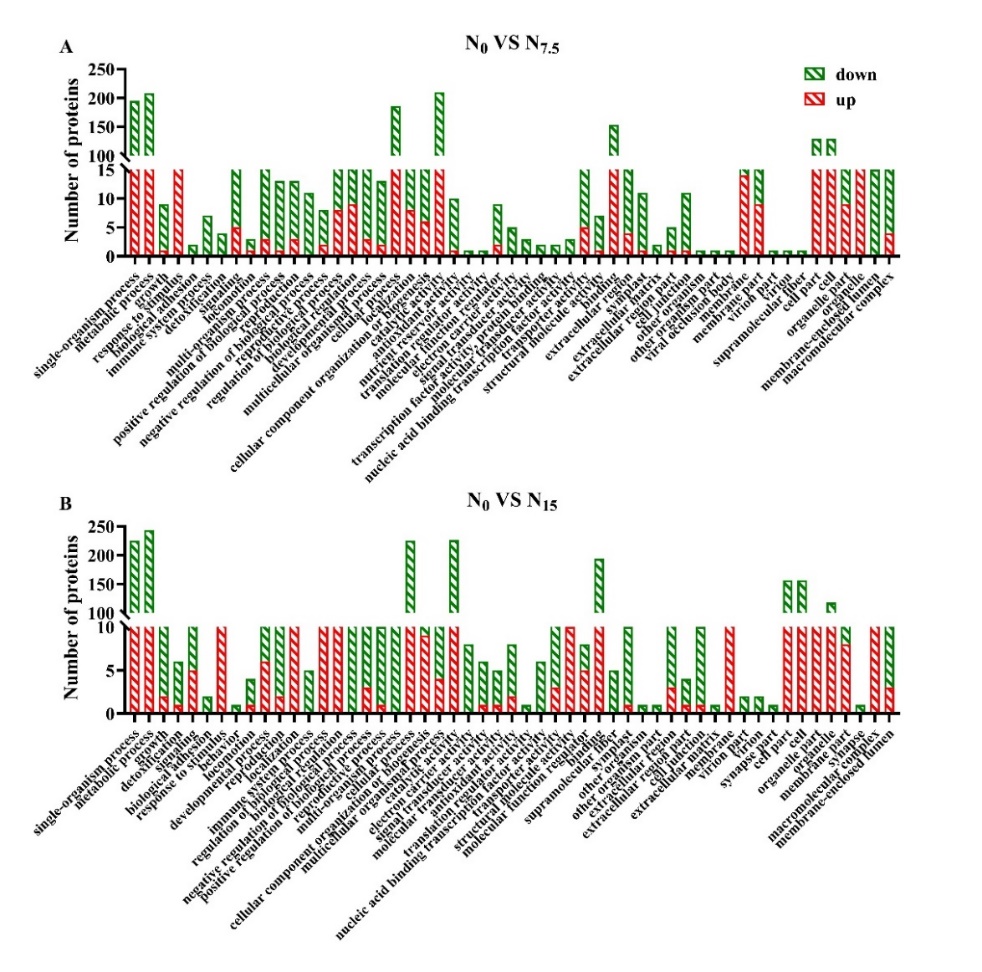


**Figure S5**


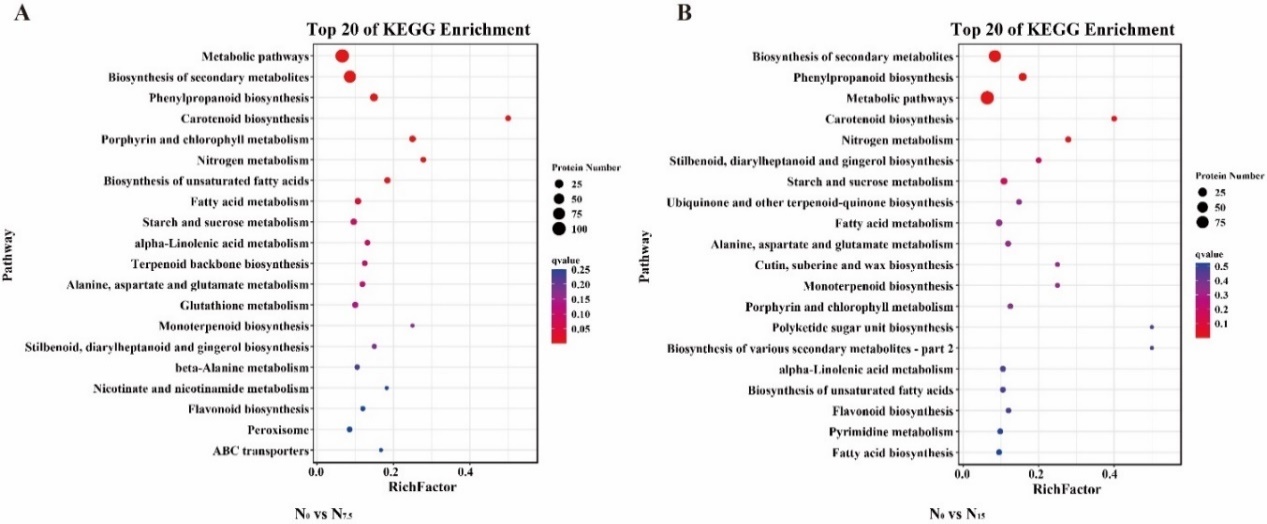


**Figure S6**


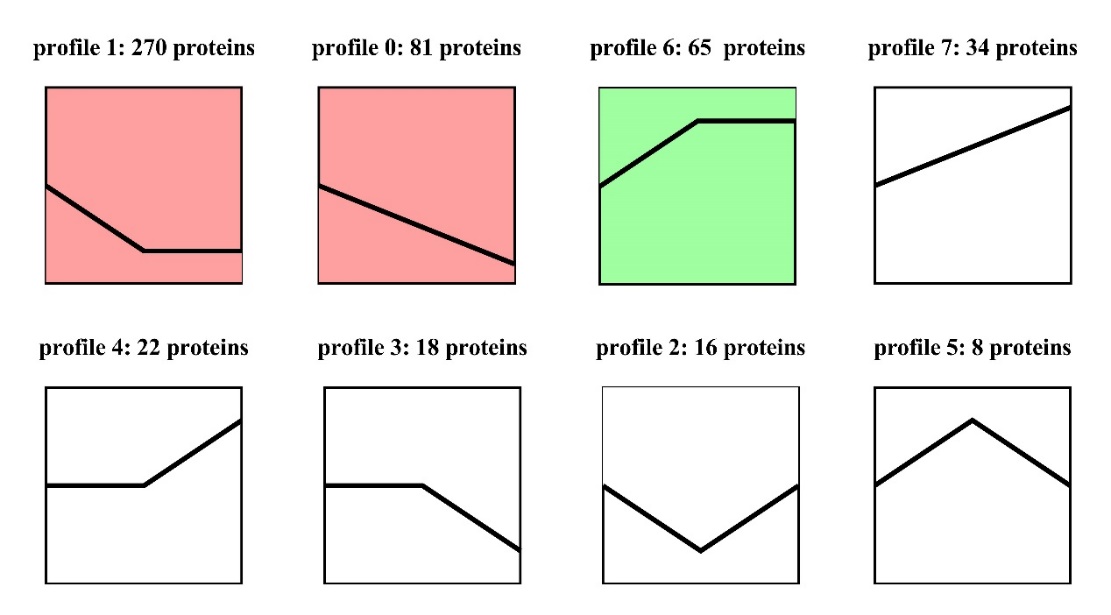


**Figure S7**


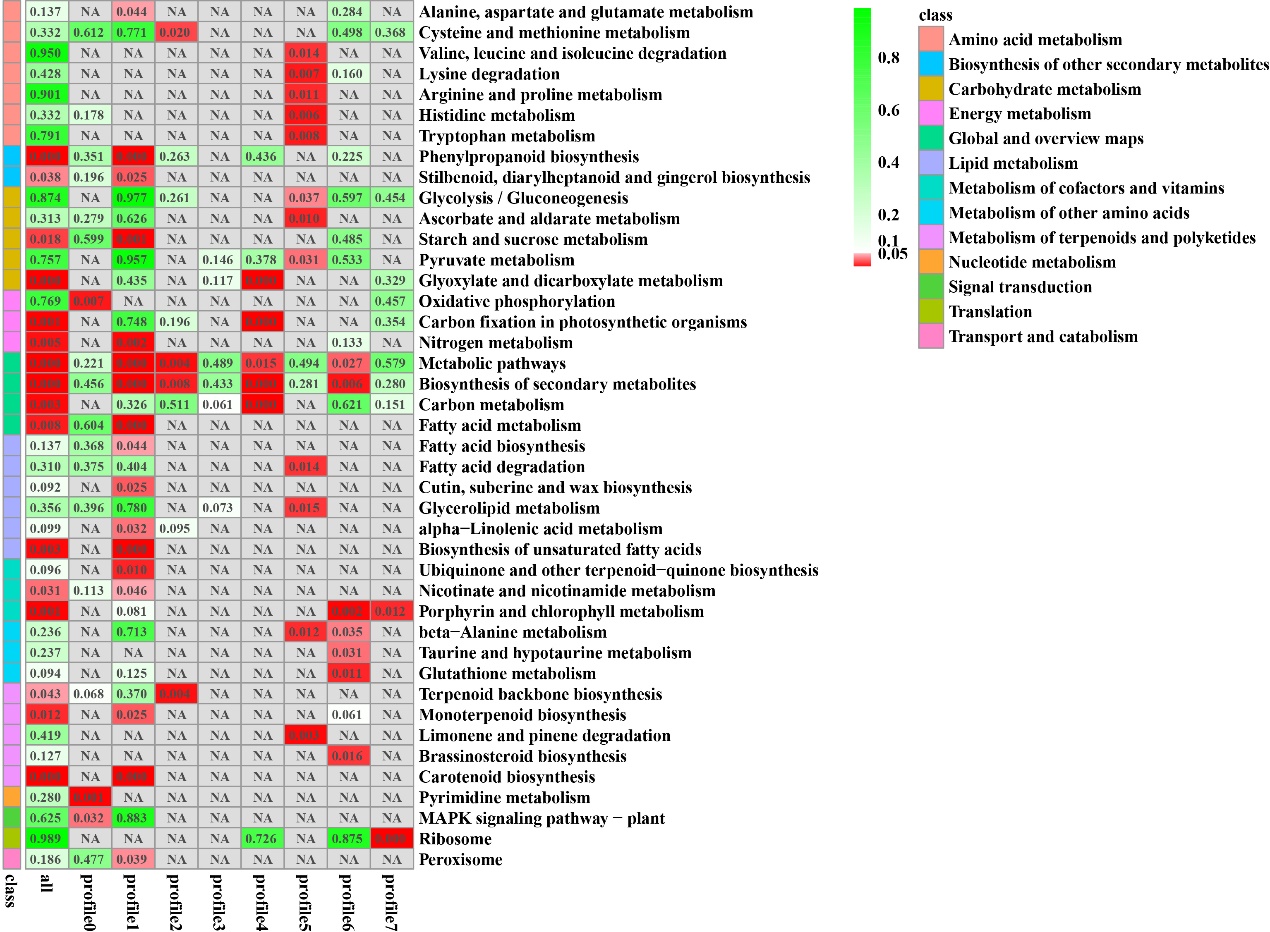


**Figure S8**


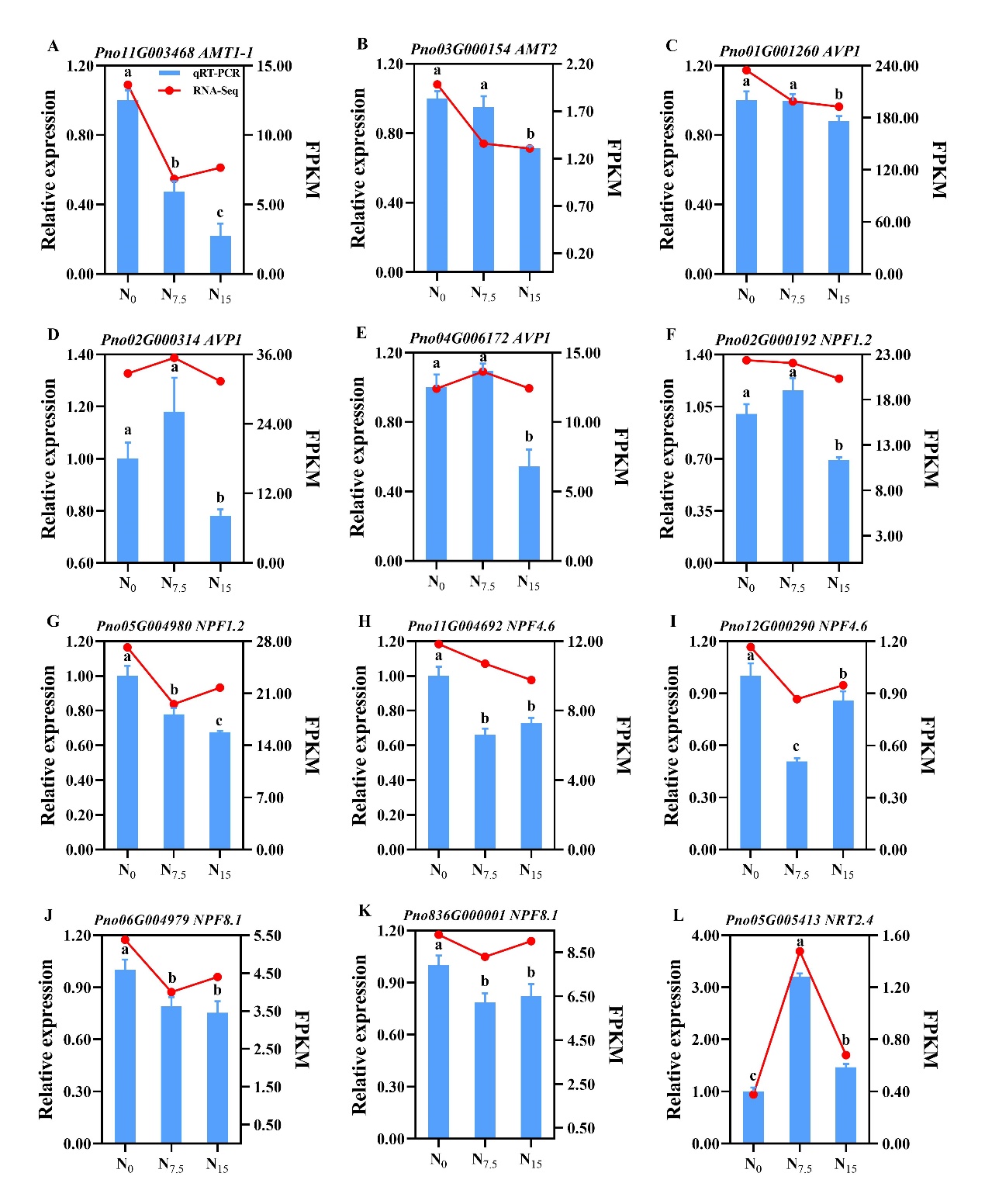


**Figure S9**


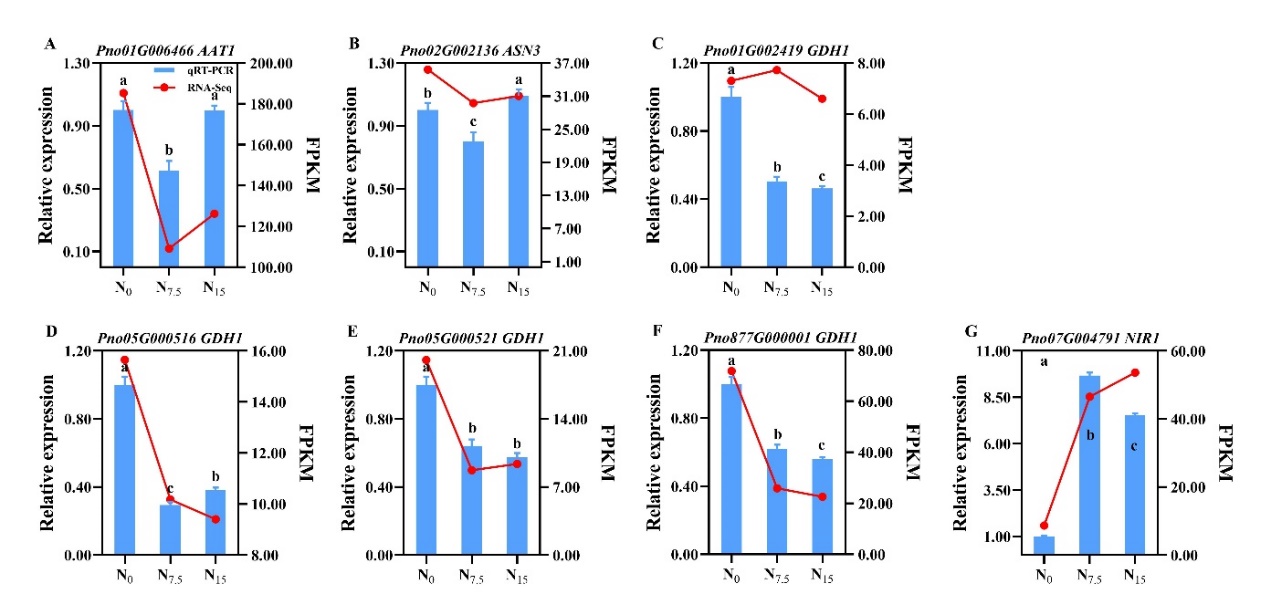


**Figure S10**


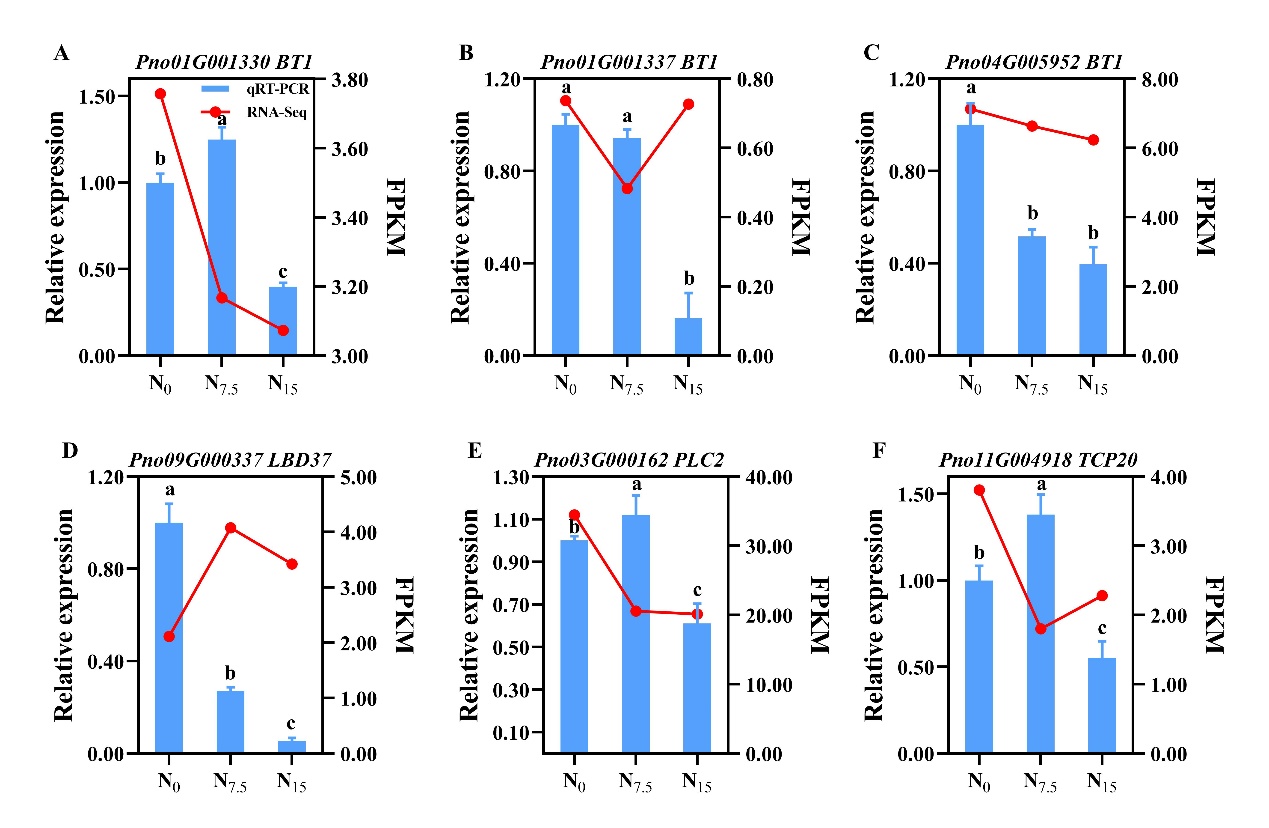


**Figure S11**


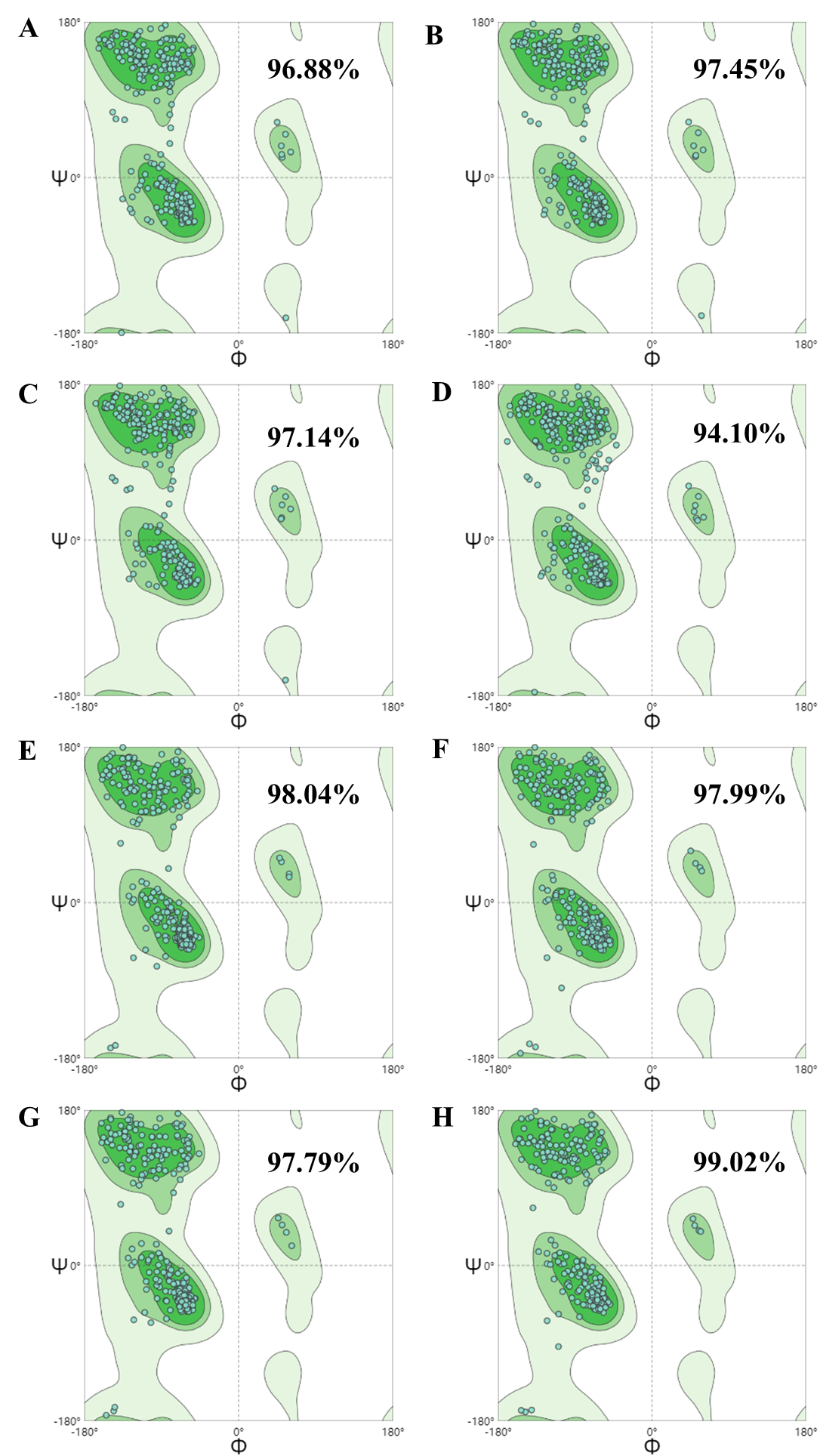


**Figure S12**


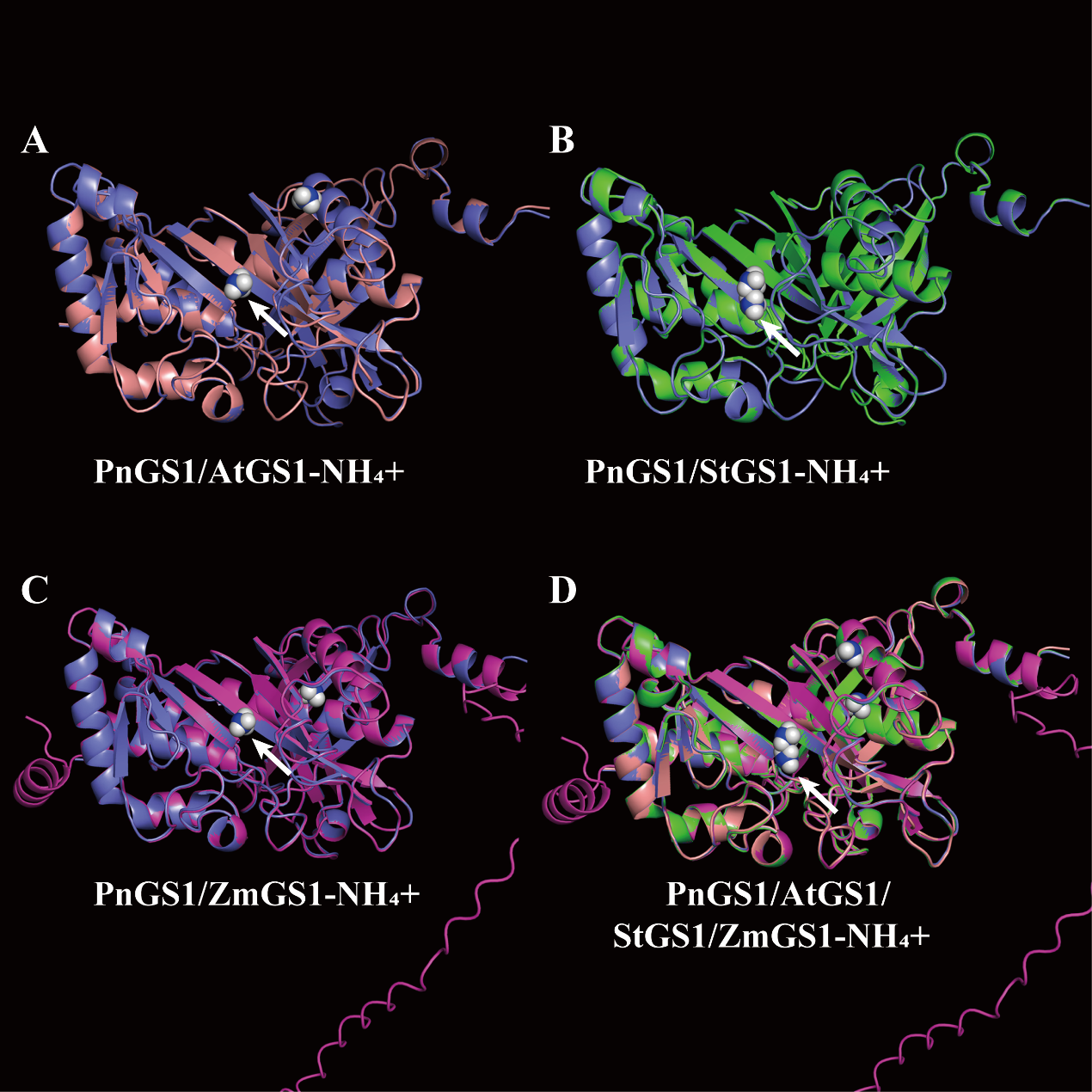


**Figure S13**


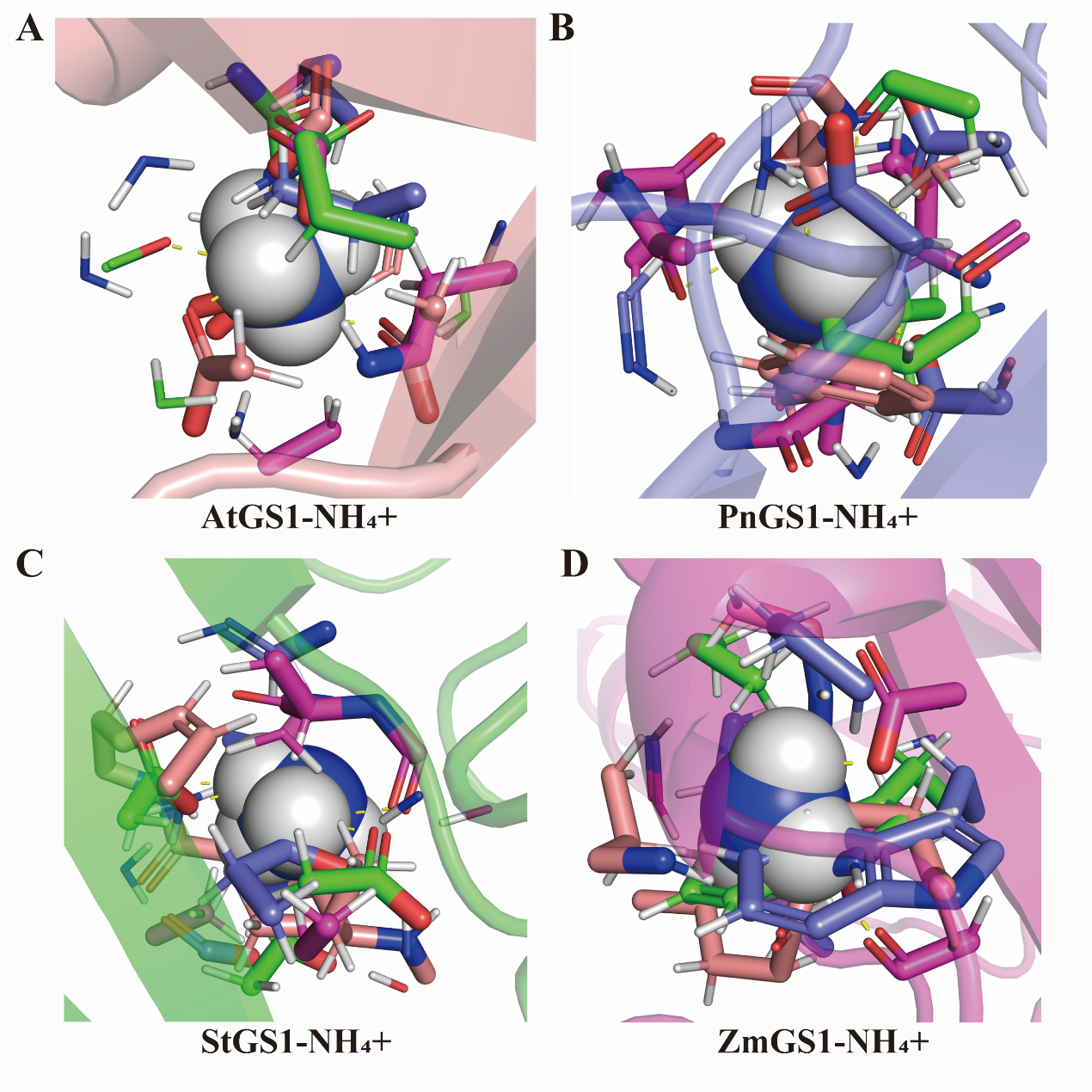


**Figure S14**


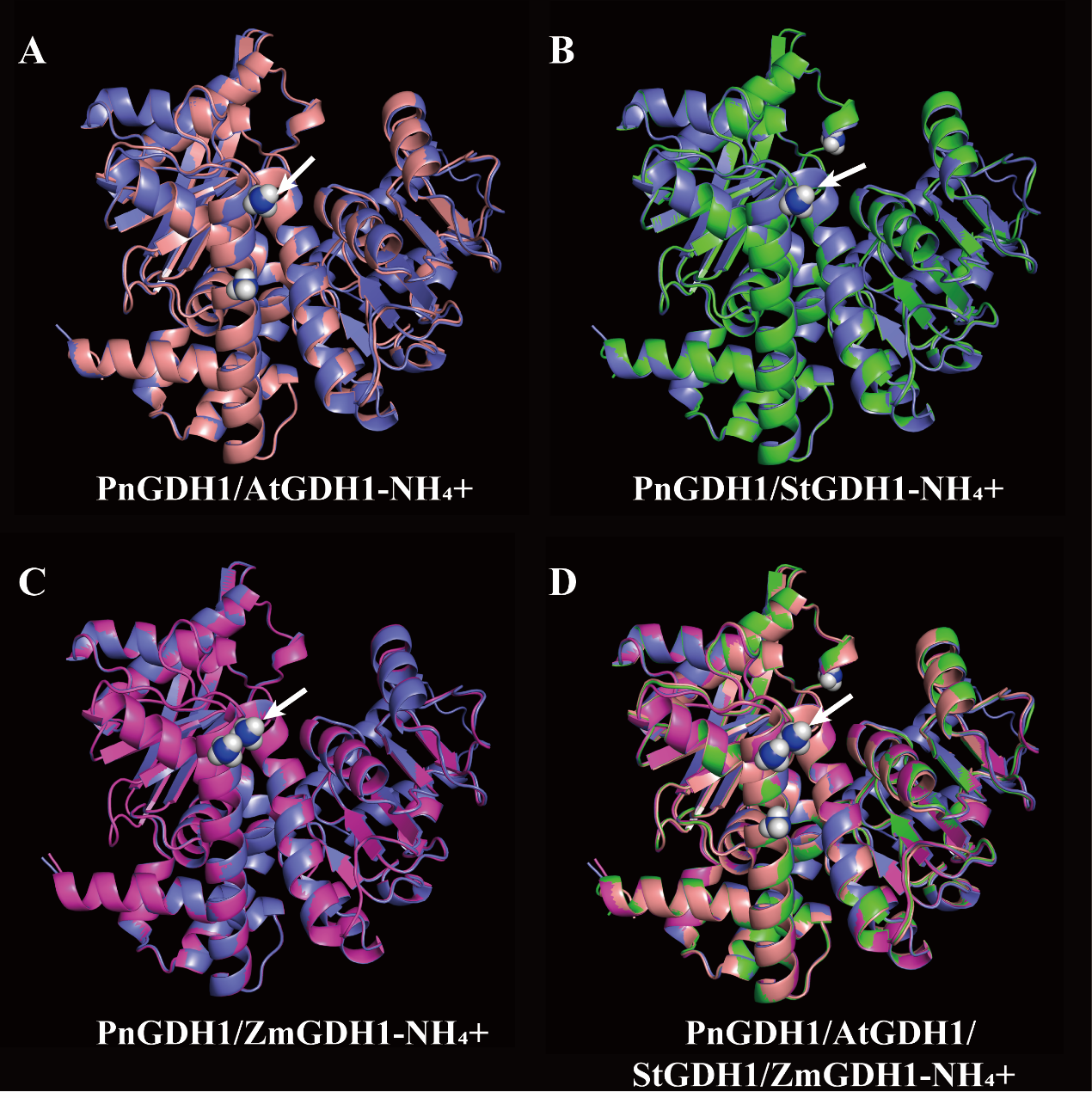


**Figure S15**


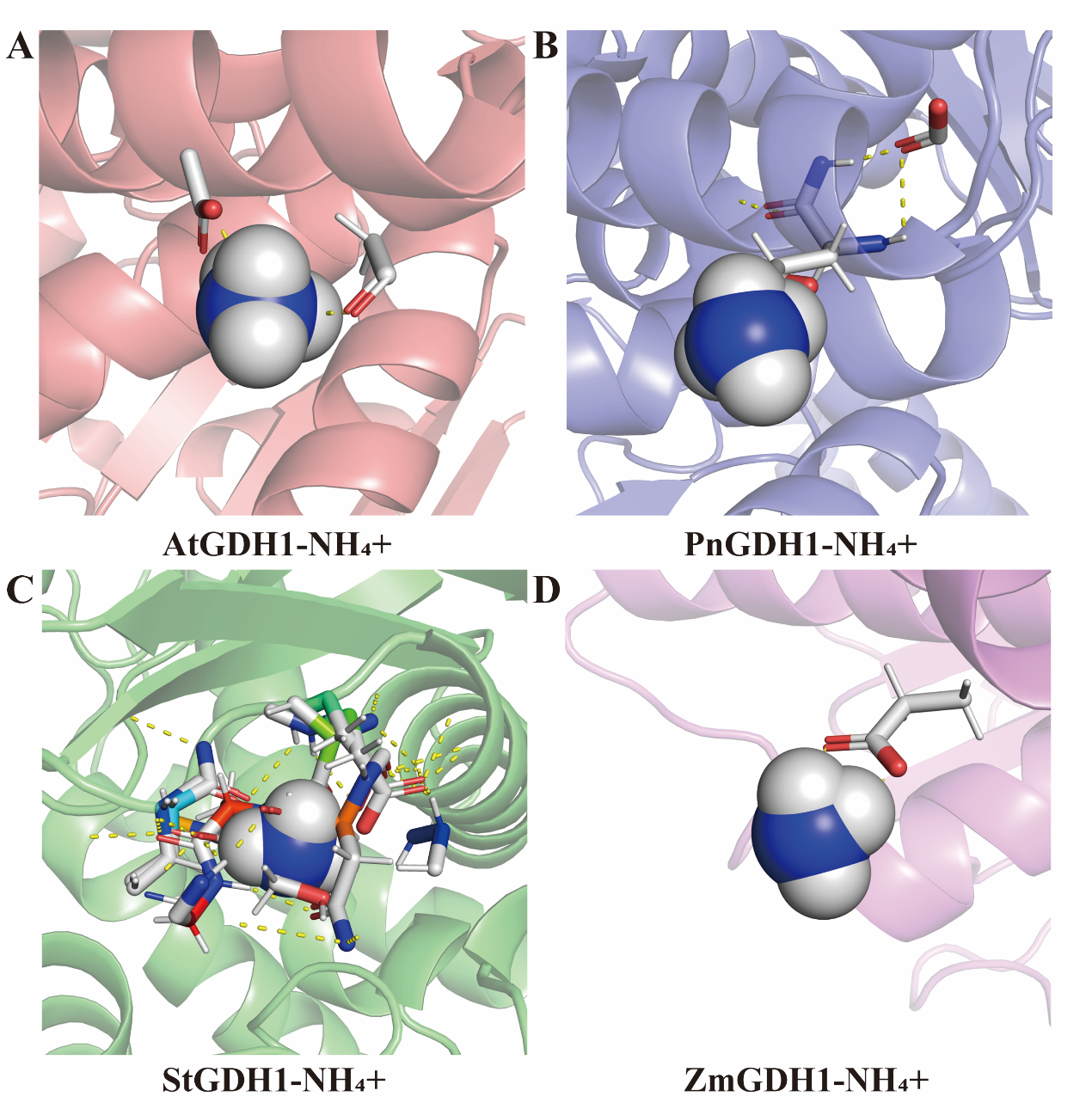

Supplement: Supplementary file 1 — Additional file 1: Figure S1. Panax notoginseng pot culture under different nitrogen (N) levels, cited from our research group (Cun et al., 2022). Figure S2. Protein (A) and peptide (B) identification. Figure S3. Annotated Venn diagrams of GO, KEGG, and KOG databases (A), comparison of differentially expressed proteins among N0, N7.5 and N15 (B). Red represents the up-regulated proteins, green represents the up-regulated proteins. Figure S4. GO analysis of differentially expressed proteins from two comparation group. (A) N0 vs N7.5, (B) N0 vs N15. Red represents the up-regulated proteins, green represents the down-regulated proteins. Figure S5. KEGG enrichment analysis for differentially expressed proteins. (A) N0 vs N7.5, (B) N0 vs N15. Red represents the up-regulated proteins, blue represents the down-regulated proteins. Figure S6. Cluster of proteins expression patterns in response to N regimes. Figure S7. Enrichment of functional categories of each cluster with the significantly enriched KEGG pathways plotted for differentially expressed proteins among N regimes. Figure S8. Real-time quantitative polymerase chain reaction (qRT-PCR) validation of key genes involved in N uptake and transport in P. notoginseng. The left Y-axis and histogram are candidate genes expression obtained via qRT-PCR, the right Y-axis and red line are gene expression level calculated as FPKM value. The relative expression obtained from real-time PCR calculated by 2−△△Ct method. Values for histogram were means ± SD (n = 3), and significant differences are indicated by letters (ANOVA; P < 0.05). The value of each red dot is the average of three biological replicates (n = 3). Figure S9. qRT-PCR validation of key genes involved in N assimilation in P. notoginseng by RNA-seq. The left Y-axis and histogram are candidate genes expression obtained via qRT-PCR, the right Y-axis and red line are gene expression level calculated as FPKM value. The relative expression obtained from real-time PCR calcul [file 12870_2024_4768_MOESM1_ESM.docx]
