# Supplementary material for: Identification of candidate genes and residues for improving nitrogen use efficiency in the N-sensitive medicinal plant Panax notoginseng
Source: BMC Plant Biol. 2024 Feb 12;24:105. doi: 10.1186/s12870-024-04768-4 (PMC10860327; doi:10.1186/s12870-024-04768-4)
Supplement: Supplementary file 2 — Additional file 2: Tabel S1. List of primers used in qRT-PCR analysis. [file 12870_2024_4768_MOESM2_ESM.docx]

**Tabel S1 List of primers used in qRT-PCR analysis.**

| Gene_Name | Forward primer | Reverse primer |
| --- | --- | --- |
| *YLS8* | CAATGACCAACCCACGACCT | TCTCGCCGAAGAAGAACGTC |
| *AMT1-1* | TCGTGTCTCACTGGTTCTGG | ACCACCAGGGAAGCACTATG |
| *AMT2* | TTTATGGTGGGAAAGGTGGA | ATGAGCCATTCCAATTACCG |
| *AVP1* | TTGCTGCAAATGGTCTTCTG | TCTGGGATGTTCCTCTCCAC |
| *AVP1-1* | GTTCTTGTGTGTGGGTGTCG | CATACATGGCAGCGAAGCTA |
| *AVP1-2* | TACGGTCCCATCAGTGACAA | AGGCACCAAATAGTGCCAAC |
| *NPF1.2* | CGATTCCTCACCATTGGACT | AATGCCGAAAGCAGAAGAAA |
| *NPF1.2-2* | GCTGAAGGTGATCCCAATGT | GGGGAGGATTAAACGGTCAT |
| *NPF4.6-1* | ATGTGACCCCAACATTCCAT | CCAGTAGCGAGGCAAAAGAC |
| *NPF4.6-2* | GCAGTCACATTGGTGGTTTG | TCTTGCTACGGCAACACAAG |
| *NPF8.1-3* | GTCTGCAGCGGTTTTAGAGG | AGCGAAAGAGCAGTGGACAT |
| *NPF8.1-5* | GGTTCGCAAAAGTCTTCGAG | AGTGTCCACCAACCAAGAGG |
| *NRT2.4-1* | TGTGGACTCTGAGCACAAGG | GGTCACAAACAGCTCCCATT |
| *NRT2.5* | GATGAGAGGGAGGTTGTGGA | TACCACTGCCCCTACAGTCC |
| *AAT1* | TTTGTGCATCAGGGATGAAA | TCCCACAACCCGTCTTTTAG |
| *ASN3* | GCGATGGAGTTGGGTACAGT | GGACCTCCTGGAACTGTTGA |
| *GDH1* | TGGGAACAAATCCACAGACA | CCGTTGTCCAGAAATGGTCT |
| *GDH1-1* | TGGGAACAAATCCACAGACA | AAACCGTTGTCCAGAAATGC |
| *GDH1-2* | TGGGAACAAATCCACAGACA | AAACCGTTGTCCAGAAATGC |
| *GDH1-3* | TGGGAACAAATCCACAGACA | AAACCGTTGTCCAGAAATGC |
| *NIR1* | GAACGGAAGATTCGGATTCA | TGTTGGGGCATTCTTTTCTC |
| *BT1* | CATGGGAGTTGGGAGAAGAA | AAGCCCTTCCATCCTTCAGT |
| *BT1-1* | CATGGGAGTTGGGAGAAGAA | AAGCCCTTCCATCCTTCAGT |
| *BT1-3* | GTGCATGAGGTGCAAGAGAA | TGTTGGGTCTTGGTTCCTTC |
| *LBD37* | CCGTCTTCGTCGCTAAGTTC | CTCTGGCATCGAAGACAACA |
| *TCP20-2* | CCTCCGTTACTGGTTTTGGA | CCGGTATGACCTTCCTGAGA |
